# Marginal interaction test for detecting interactions between genetic marker sets and environment in genome-wide studies

**DOI:** 10.1093/g3journal/jkae263

**Published:** 2024-11-14

**Authors:** Linchuan Shen, Amei Amei, Bowen Liu, Gang Xu, Yunqing Liu, Edwin C Oh, Xin Zhou, Zuoheng Wang

**Affiliations:** Department of Mathematical Sciences, University of Nevada, Las Vegas, Las Vegas, NV 89154, USA; Department of Mathematical Sciences, University of Nevada, Las Vegas, Las Vegas, NV 89154, USA; Nevada Institute of Personalized Medicine, University of Nevada, Las Vegas, Las Vegas, NV 89154, USA; Department of Mathematical Sciences, University of Nevada, Las Vegas, Las Vegas, NV 89154, USA; Division of Computing, Analysis, and Mathematics, University of Missouri, Kansas City, MO 64108, USA; Department of Mathematical Sciences, University of Nevada, Las Vegas, Las Vegas, NV 89154, USA; Department of Biostatistics, Yale School of Public Health, Yale University, New Haven, CT 06510, USA; Department of Biostatistics, Yale School of Public Health, Yale University, New Haven, CT 06510, USA; Nevada Institute of Personalized Medicine, University of Nevada, Las Vegas, Las Vegas, NV 89154, USA; Department of Internal Medicine, University of Nevada School of Medicine, Las Vegas, NV 89154, USA; Department of Biostatistics, Yale School of Public Health, Yale University, New Haven, CT 06510, USA; Department of Biostatistics, Yale School of Public Health, Yale University, New Haven, CT 06510, USA; Department of Biomedical Informatics and Data Science, Yale School of Medicine, Yale University, New Haven, CT 06510, USA

**Keywords:** gene–environment interaction, genome-wide study, method of moments, mixed effects model

## Abstract

As human complex diseases are influenced by the interaction between genetics and the environment, identifying gene–environment interactions (G×E) is crucial for understanding disease mechanisms and predicting risk. Developing robust quantitative tools for G×E analysis can enhance the study of complex diseases. However, many existing methods that explore G×E focus on the interplay between an environmental factor and genetic variants, exclusively for common or rare variants. In this study, we developed MAGEIT_RAN and MAGEIT_FIX to identify interactions between an environmental factor and a set of genetic markers, including both rare and common variants, based on the MinQue for Summary statistics. The genetic main effects in MAGEIT_RAN and MAGEIT_FIX are modeled as random and fixed effects, respectively. Simulation studies showed that both tests had type I error under control, with MAGEIT_RAN being the most powerful test. Applying MAGEIT to a genome-wide analysis of gene–alcohol interactions on hypertension and seated systolic blood pressure in the Multiethnic Study of Atherosclerosis revealed genes like *EIF2AK2*, *CCNDBP1*, and *EPB42* influencing blood pressure through alcohol interaction. Pathway analysis identified 1 apoptosis and survival pathway involving PKR and 2 signal transduction pathways associated with hypertension and alcohol intake, demonstrating MAGEIT_RAN's ability to detect biologically relevant gene–environment interactions.

## Introduction

The causes of human common diseases are multifactorial, involving a complex interplay between genetic and environmental factors. The effect of environmental exposures on disease outcomes can vary across different genotypic groups. In many common diseases, individuals with specific genetic profiles may only experience increased disease risk when exposed to certain environmental factors (Lin *et al.* 2013). For instance, various environmental factors such as smoking, drinking, diet, stress, and air quality can influence disease risk, progression, and severity (Cosselman *et al.* 2015; Bhatnagar 2017). Consequently, incorporating gene–environment interactions (G×E) has become essential in the study of complex traits. Genome-wide association studies (GWAS) have successfully identified numerous common genetic variants with a minor allele frequency (MAF) greater than 0.05 that are associated with human diseases. However, the estimated effects of these variants are modest, accounting for only a small fraction of the heritability observed in common diseases (Eichler *et al.* 2010; Bass *et al.* 2024). Gene–environment interactions are increasingly recognized as potential contributors to the missing heritability (Bass *et al.* 2024; Herrera-Luis *et al.* 2024). Recent studies emphasized that these interactions can significantly modulate the impact of genetic variants on traits and diseases, accounting for some of the unexplained genetic influences (Bass *et al.* 2024).

Traditional G×E analyses have generally focused on assessing interactions with genetic variants one at a time (Kraft *et al.* 2007; Aschard *et al.* 2010; Manning *et al.* 2011). However, this approach has potential limitations, such as the challenge of multiple hypothesis testing and the inability to consider the combined effects of multiple variants with related biological functions, which can lead to reduced statistical power (Lin *et al.* 2013). In recent years, genome-wide search for G×E has been emerging (Khoury and Wacholder 2009; Thomas 2010). Several studies have investigated G×E by analyzing multiple genetic variants within a marker set (Tzeng *et al.* 2011; Jiao *et al.* 2013; Lin *et al.* 2013; Chen *et al.* 2014; Lin *et al.* 2016; Su *et al.* 2017; Lin *et al.* 2019; Wang *et al.* 2020; Chi *et al.* 2021). For common genetic variants, the gene–environment set association test (GESAT) was developed using a generalized linear model and ridge regression (Lin *et al.* 2013). For rare variants, Chen *et al.* proposed INT-FIX and INT-RAN to assess G×E effects, along with a joint test called JOINT, which simultaneously detects the effects of a set of genetic variants and their interactions with an environmental factor (Chen *et al.* 2014). These tests model genetic effects using a beta density function, accounting for the larger contributions from rare variants. In INT-FIX, genetic main effects are treated as fixed when the environmental factor is absent, while in INT-RAN, they are treated as random. To evaluate rare variant–environment interactions, Lin *et al.* developed the interaction sequence kernel association test (iSKAT), which models the main effects of rare variants using weighted ridge regression and considers correlations in interactions with the environment across genetic variants (Lin *et al.* 2016). These variance component-based tests are robust when many variants in a genetic region are noncausal and/or exhibit mixed beneficial and detrimental effects (Wu *et al.* 2011; Lee *et al.* 2012; Santorico and Hendricks 2016; Su *et al.* 2017). Subsequently, Su *et al.* developed the mixed effects score test for interaction (MiSTi), a unified regression framework for testing interactions between a set of rare variants and an environmental factor (Su *et al.* 2017). Many existing G×E testing methods can be derived from MiSTi by setting certain parameters to zero. Additionally, Lin *et al.* proposed the adaptive combination of Bayes factors (ADABF) method, a polygenic test of G×E effects using Bayes factors, which assumes that G×E effects follow a normal distribution (Lin *et al.* 2019). In this method, variants in a genetic region are ranked by Bayes factors, and *P*-values are calculated through resampling. While ADABF considers both common and rare variants within a genetic region, it does not distinguish between their effects in the model, potentially overlooking the larger contributions from rare variants (Wu *et al.* 2011; Chen *et al.* 2022).

In this study, we developed 2 tests to detect interactions between an environmental factor and a set of genetic markers that include both rare and common variants. These tests are based on the variance component estimation method MinQue for Summary statistics (MQS) (Zhou 2017). The strength of MQS lies in its ability to provide unbiased and statistically efficient estimates using the method of moments and the minimal norm quadratic unbiased estimation criterion. We named these 2 tests the MArginal Gene–Environment Interaction Test with RANdom or FIXed genetic effects (MAGEIT_RAN and MAGEIT_FIX), respectively. Compared to existing methods, MAGEIT_RAN and MAGEIT_FIX not only incorporate both common and rare variants within a genetic region but also differentiate their effects by assigning different weights to the coefficients of the common and rare genetic variants during model fitting. Additionally, they produce unbiased estimators for the variance components.

To assess the performance of these tests in detecting G×E interactions for a set of genetic variants, we conducted simulation studies and compared them with existing set-based G×E methods. Our results showed that both MAGEIT_RAN and MAGEIT_FIX effectively controlled type I error, with MAGEIT_RAN consistently exhibiting the highest statistical power across all simulation scenarios. Furthermore, we applied MAGEIT_RAN and MAGEIT_FIX to a genome-wide analysis of gene–alcohol interactions with hypertension and seated systolic blood pressure (SBP) in the Multiethnic Study of Atherosclerosis (MESA). Using SBP as a continuous phenotype, MAGIT_RAN identified 1 gene with significant gene–alcohol interactions at the genome-wide level. When analyzing hypertension as a binary trait, MAGIT_RAN identified 2 genes with gene–alcohol interactions at the smallest *P*-values among the G×E tests compared. Furthermore, we discovered 3 pathways associated with SBP, hypertension, and alcohol consumption.

## Materials and methods

Suppose a phenotype of interest, an environmental variable and genome-wide genetic variants are available on *n* subjects. The genotype of a variant can be directly measured or imputed values. Let $y_{k},E_{k},G_{k}=(G_{k1},\;G_{k2},\;\ldots ,G_{kp}{)}^{T},$ and $X_{k}=(X_{k1},\;X_{k2},\;\ldots ,X_{km}{)}^{T}$ denote the phenotype, environmental variable, genotypes of *p* variants in a genomic region, and *m* nongenetic covariates for the *k* th subject, respectively, for k=1,2,…,n, where $G_{kj}=$ 0, 1, or 2 depending on whether subject *k* has 0, 1, or 2 copies of minor allele at the *j*th variant. We use $S_{k}=(E_{k}G_{k1},\;E_{k}G_{k2},\;\ldots ,E_{k}G_{kp}{)}^{T}$ to denote the genetic variants by environment interaction for the *k*th subject. Our goal is to test whether there are interactions between the variant set and the environment that influence the phenotype of interest.

### Model for continuous phenotype

Let $y=(y_{1},\;y_{2},\;\ldots ,\;y_{n}{)}^{T}$, $E=(E_{1},\;E_{2},\;\ldots ,E_{n}{)}^{T}$, and $\varepsilon =(\varepsilon_{1},\varepsilon_{2},\cdots ,\varepsilon_{n}{)}^{T}$ denote vectors of the phenotype, environmental variable, and error term of length *n*, respectively. We further define an n×m covariate matrix $X=[X_{1},X_{2},\cdots ,X_{n}{]}^{T}$, an n×p genotype matrix $G=[G_{1},\;G_{2},\;\cdots ,G_{n}{]}^{T}$, and an n×p matrix $S=[S_{1},S_{2},\cdots ,S_{n}{]}^{T}$ of the G×E. Then, the following model specifies the relationship between a continuous phenotype Y and X,E,G, and S


(1) $$y=\alpha_{0}1+X\alpha_{1}+\alpha_{2}E+G\beta +S\gamma +\varepsilon ,$$

where **1** is an n×1 vector of 1, $\alpha_{0}$ is an intercept term, $\alpha_{1}=(\alpha_{11},\;\alpha_{12},\;\ldots ,\;\alpha_{1m}{)}^{T}$, $\alpha_{2}$, $\beta =(\beta_{1},\;\beta_{2},\;\ldots ,\;\beta_{p}{)}^{T}$, and $\gamma =(\gamma_{1},\;\gamma_{2},\;\ldots ,\;\gamma_{p}{)}^{T}$ are regression coefficients for the covariates, environmental factor, genetic variants, and G×E terms, respectively. We further assume that γ and ε follow multivariate normal distributions with $\gamma \;∼\;\mathrm{MVN}\left(0,\;\frac{\sigma^{2}}{p}W_{2}^{2}\right)$ and $\varepsilon \;∼\;\mathrm{MVN}(0,\;\tau^{2}I_{n})$, where $W_{2}=\mathrm{diag}(w_{21},w_{22},\cdots ,w_{2p})$ contains weights of the *p*  G×E terms and $I_{n}$ is an identity matrix of dimension *n*.

### Marginal gene–environment interaction test

We are interested in testing genetic variants by environment interactions in a genomic region, i.e. testing the null hypothesis $H_{0}:\;\gamma =0$, which is equivalent to testing $H_{0}:\;\sigma^{2}=0$. We develop 2 G×E tests, in which the genetic main effects β are modeled as random or fixed effects, respectively.

When we treat the genetic main effects β as random, we assume that $\beta ∼\;\mathrm{MVN}\left(0,\;\frac{\omega^{2}}{p}W_{1}^{2}\right)$, where $W_{1}=\mathrm{diag}(w_{11},w_{12},\cdots ,w_{1p})$ are weights of the *p* variants. We use the MQS method (Zhou 2017) to estimate the 3 variance components $\omega^{2}$, $\sigma^{2}$, and $\tau^{2}$. In order to eliminate the fixed effects $\alpha_{0},\alpha_{1}$, and $\alpha_{2}$ in Model (1), we multiply both sides of the model, from left, by a projection matrix M, where $M=I-b(b^{T}b{)}^{-1}b^{T}$ with b=[1,X,E]. Then Model (1) becomes


$$y^{*}=g^{*}+s^{*}+\varepsilon^{*},$$

where $y^{*}=My$, $g^{*}=MG\beta$, $s^{*}=MS\gamma$, and $\varepsilon^{*}=M\varepsilon$. It follows that $g^{*}\;∼\;\mathrm{MVN}(0,\;\omega^{2}G^{*})$ with $G^{*}=\frac{(MGW_{1}){\left(MGW_{1}\right)}^{T}}{p}$, $s^{*}\;∼\;\mathrm{MVN}(0,\;\sigma^{2}S^{*})$ with $S^{*}=\frac{(MSW_{2}){\left(MSW_{2}\right)}^{T}}{p}$, and $\varepsilon^{*}\;∼\;\mathrm{MVN}(0,\tau^{2}M)$. Consequently, we have $y^{*}\;∼\;\mathrm{MVN}(0,\;\omega^{2}G^{*}+\sigma^{2}S^{*}+\tau^{2}M)$.

We estimate the variance components using the method of moments based on the following set of second moment matching equations


(2) $$\begin{array}{rl} E(y^{*T}Ay^{*})\;= & \mathrm{tr}(A(\omega^{2}G^{*}+\sigma^{2}S^{*}+\tau^{2}M))=\omega^{2}\mathrm{tr}(AG^{*})+\sigma^{2}\mathrm{tr}(AS^{*})\\ & +\tau^{2}\mathrm{tr}(AM), \end{array}$$

where A is an arbitrary symmetric non-negative definite matrix (Zhou 2017). Since there are 3 unknown parameters $\left(\omega^{2},\sigma^{2},\tau^{2}\right)$, 3 different A ’s are required to obtain parameter estimates. In the method of moments, the expectation of Eq. (2) is usually replaced with the realized value $y^{*T}Ay^{*}$. Let $A_{1}=G^{*}$, $A_{2}=S^{*}$, and $A_{3}=M$ (Zhou 2017); then, the resulting estimates of the variance components are given in a matrix form as


$$\begin{array}{rl} \left[\begin{array}{c} {\hat{\omega }}^{2}\\ \begin{array}{c} {\hat{\sigma }}^{2}\\ {\hat{\tau }}^{2} \end{array} \end{array}\right] & =\Lambda^{-1}\left[\begin{array}{c} y^{*T}G^{*}y^{*}\\ y^{*T}S^{*}y^{*}\\ y^{*T}y^{*} \end{array}\right]\\ & =\;{\left[\begin{array}{c} \begin{array}{ccc} \mathrm{tr}(G^{*}G^{*}) & \mathrm{tr}(G^{*}S^{*}) & tr(G^{*}) \end{array}\\ \begin{array}{ccc} \mathrm{tr}(S^{*}G^{*}) & \mathrm{tr}(S^{*}S^{*}) & tr(S^{*}) \end{array}\\ \begin{array}{ccc} \mathrm{tr}(G^{*}) & \mathrm{tr}(S^{*}) & n-(m+2) \end{array} \end{array}\right]}^{-1}\left[\begin{array}{c} y^{*T}G^{*}y^{*}\\ y^{*T}S^{*}y^{*}\\ y^{*T}y^{*} \end{array}\right], \end{array}$$

where we used $\mathrm{tr}(G^{*}M)=\mathrm{tr}(MG^{*})=\mathrm{tr}(G^{*})$, $\mathrm{tr}(S^{*}M)=\mathrm{tr}(MS^{*})=\mathrm{tr}(S^{*})$, tr(MM)=tr(M)=n−(m+2), and $y^{*T}My^{*}=y^{*T}y^{*}$. The variance component estimator ${\hat{\sigma }}^{2}$ is considered as the test statistic, which we named as MArginal Gene–Environment Interaction Test with RANdom genetic main effects (MAGEIT_RAN). Specifically, the MAGEIT_RAN test statistic is


(3) $${\hat{\sigma }}^{2}=y^{*T}\{{\left(\Lambda^{-1}\right)}_{21}G^{*}+{\left(\Lambda^{-1}\right)}_{22}S^{*}+{\left(\Lambda^{-1}\right)}_{23}I\}y^{*}=y^{*T}Hy^{*},$$

where $H=(\Lambda^{-1}{)}_{21}G^{*}+(\Lambda^{-1}{)}_{22}S^{*}+(\Lambda^{-1}{)}_{23}I$.

Under $H_{0}:\;\sigma^{2}=0$, $y^{*}\;∼\;\mathrm{MVN}(0,\;\omega^{2}G^{*}+\tau^{2}M)$, suggesting that $y^{*}$ has the same distribution as $(\omega^{2}G^{*}+\tau^{2}M{)}^{\frac{1}{2}}Z$ with $Z\;∼\;\mathrm{MVN}(0,I_{n})$. Therefore, the method of moments estimator ${\hat{\sigma }}^{2}$ follows the same distribution as $Z^{T}{\left({\left({\hat{\omega }}_{0}^{2}G^{*}+{\hat{\tau }}_{0}^{2}M\right)}^{\frac{1}{2}}\right)}^{T}H({\hat{\omega }}_{0}^{2}G^{*}+{\hat{\tau }}_{0}^{2}M{)}^{\frac{1}{2}}Z$, which has a mixture of $\chi^{2}$ distribution ${\hat{\sigma }}^{2}∼\overset{n}{\underset{i=1}{\sum }}\;\lambda_{i}\chi_{1,i}^{2}$. Here, $\left({\hat{\omega }}_{0}^{2},\;{\hat{\tau }}_{0}^{2}\right)$ are estimates of $\left(\omega^{2},\tau^{2}\right)$ under the null hypothesis, $\left(\lambda_{1},\cdots ,\lambda_{n}\right)$ are eigenvalues of the matrix ${\left({\left({\hat{\omega }}_{0}^{2}G^{*}+{\hat{\tau }}_{0}^{2}M\right)}^{\frac{1}{2}}\right)}^{T}H({\hat{\omega }}_{0}^{2}G^{*}+{\hat{\tau }}_{0}^{2}M{)}^{\frac{1}{2}}$, and $\chi_{1,i}^{2}$ are independent $\chi_{1}^{2}$ variables (Zhou 2017). The *P*-value of ${\hat{\sigma }}^{2}$ can be evaluated by the Davies method (Davies 1980; Wu *et al.* 2011) and Liu–Tang–Zhang approximation (Liu *et al.* 2009).

If we treat the genetic main effects β as fixed, we use the MQS method (Zhou 2017) to estimate the 2 variance components $\sigma^{2}$ and $\tau^{2}$. To eliminate the fixed effect terms $\alpha_{0},\alpha_{1}$, $\alpha_{2}$, and β in Model (1), we left multiply the model by a projection matrix $M=I-b(b^{T}b{)}^{-1}b^{T}$ with b=[1,X,E,G]. Then the model becomes $y^{*}=s^{*}+\;\varepsilon^{*}$, and it contains 2 variance components $\sigma^{2}$ and $\tau^{2}$. Using the method of moments, we obtain the following estimates of the variance components


$$\left[\begin{array}{c} {\hat{\sigma }}^{2}\\ {\hat{\tau }}^{2} \end{array}\right]=\;{\left[\begin{array}{c} \begin{array}{cc} tr(S^{*}S^{*}) & tr(S^{*}) \end{array}\\ \begin{array}{cc} tr(S^{*}) & n-(m+p+2) \end{array} \end{array}\right]}^{-1}\left[\begin{array}{c} y^{*T}S^{*}y^{*}\\ y^{*T}y^{*} \end{array}\right].$$

The variance component estimator ${\hat{\sigma }}^{2}$ is considered as the test statistic, which we named as MArginal Gene–Environment Interaction Test with FIXed genetic main effects (MAGEIT_FIX). Specifically, the MAGEIT_FIX test statistic is


(4) $${\hat{\sigma }}^{2}=\frac{y^{*T}\{(n-(m+p+2))S^{*}-tr(S^{*})I\}y^{*}}{(n-(m+p+2))tr(S^{*}S^{*})-tr{\left(S^{*}\right)}^{2}}.$$

Under $H_{0}:\;\sigma^{2}=0$, ${\hat{\sigma }}^{2}$ follows a mixture of $\chi^{2}$ distribution ${\hat{\sigma }}^{2}∼\overset{n}{\underset{i=1}{\sum }}\;\lambda_{i}\chi_{1,i}^{2}$ with $\left(\lambda_{1},\cdots ,\lambda_{n}\right)$ being the eigenvalues of the matrix ${\left({\left({\hat{\tau }}_{0}^{2}M\right)}^{\frac{1}{2}}\right)}^{T}H({\hat{\tau }}_{0}^{2}M{)}^{\frac{1}{2}}$.

### Model for binary phenotype

We consider a liability threshold model and assume the binary outcome $y_{k}$ of the *k* th subject is determined by an unobserved continuous liability variable $z_{k}$, i.e.


(5) $$y_{k}=\{\begin{array}{c} 1,\;\;z_{k}\geq 0\\ 0,\;\;z_{k}<0 \end{array}\;\;\;\;\;\;\;\mathrm{for}\;k=1,\;\ldots ,\;n,$$

where the underlying liability vector $z=(z_{1},\;z_{2},\cdots ,z_{n}{)}^{T}$ is specified using Model (1). The full likelihood of the liability threshold mixed effects model is intractable due to an *n*-dimensional integration over the liability variable z. Following previous studies (Tempelman and Gianola 1993; Engel *et al.* 1995; Williams and Barber 1998; Kuss *et al.* 2005; Crawford and Zhou 2018), the liability threshold mixed effects model can be approximated by a linear mixed effects model on $\hat{z}=E(z|y)$, an estimated posterior mean of the liabilities


(6) $$\hat{z}=\alpha_{0}1+X\alpha_{1}+\alpha_{2}E+G\beta +S\gamma +\varepsilon .$$

The posterior mean $\hat{z}$ can be obtained by approximation under certain assumptions based on the properties of GWAS data (Crawford and Zhou 2018). Specifically, we assume that (1) subjects are unrelated and (2) both the genetic main effects and interaction effects are small such that the terms Gβ and Sγ can be ignored. Under these assumptions, the distribution of the liability variable can be approximated by $z∼\mathrm{MVN}(\alpha_{0}1+X\alpha_{1}+\alpha_{2}E,\;I_{n})$, and $\hat{z}$ is computed as the mean of the following truncated normal distribution (Crawford and Zhou 2018):


$$z_{k}|y_{k}\;∼\;\{\begin{array}{c} \begin{array}{ccc} N(\alpha_{0}+X_{k}^{T}\alpha_{1}+\alpha_{2}E_{k},1) & \mathrm{with}\;z_{k}\geq 0 & \mathrm{if}\;y_{k}=1 \end{array}\\ \begin{array}{ccc} N(\alpha_{0}+X_{k}^{T}\alpha_{1}+\alpha_{2}E_{k},1) & \mathrm{with}\;z_{k}<0 & \mathrm{if}\;y_{k}=0 \end{array} \end{array}\;\mathrm{for}\;k=1,\;2,\;\ldots ,\;n.$$

The parameters $\alpha_{0}$,$\alpha_{1}$, and $\alpha_{2}$ are estimated using a probit model on the phenotype y.

To test the interaction effects between a set of genetic variants and an environmental variable on the binary phenotype y, we implement MAGEIT_RAN or MAGEIT_FIX on the estimate of the liability variable $\hat{z}$. To construct MAGEIT_RAN, the liability threshold mixed effects model specified in Eqs. (5) and (6) contains 3 variance components $\left(\omega^{2},\sigma^{2},\tau^{2}\right)$, where $\sigma^{2}$ represents a measure of interactions between the *p* genetic variants and the environmental variable. In order for the model to be identifiable, we put a constraint on the variance of z, e.g. $\omega^{2}+\sigma^{2}+\tau^{2}=1$ (Lee *et al.* 2011). Similarly, we set $\sigma^{2}+\tau^{2}=1$ for MAGEIT_FIX.

### Simulation studies

We performed simulation studies to assess the effectiveness of MAGEIT_RAN and MAGEIT_FIX in detecting set-based G×E interactions for both continuous and binary phenotypes, where the variant set included both common and rare variants. We evaluated the type I error and empirical power of MAGEIT_RAN and MAGEIT_FIX, comparing them with 3 established set-based G×E tests: GESAT-W (Lin *et al.* 2013), aMiSTi (Su *et al.* 2017), and ADABF (Lin *et al.* 2019). These 3 methods are frequently used in G×E analysis and are supported by well-developed R packages. To ensure a fair comparison, identical weights for rare and common variants were applied across all methods, except for ADABF, which does not differentiate between common and rare variants and therefore did not utilize weights in its implementation.

For the gene sets, we selected 2 genes of different lengths from chromosome 22 in the MESA data. The first, *A4GALT*, contains 200 variants, including 38 (19%) common (MAF > 0.05) and 162 (81%) rare (0.005 < MAF < 0.05) variants. The second, *TIMP3*, has 296 variants, with 107 (36%) common and 189 (64%) rare variants.

### Trait models

We simulated a continuous phenotype using the following trait model


$$y_{k}=0.05X_{k1}+0.057X_{k2}+0.64E_{k}+\overset{10}{\underset{\;j=1}{\sum }}\;w_{1j}\beta_{j}G_{kj}+\overset{10}{\underset{l=1}{\sum }}\;w_{2l}\gamma_{l}E_{k}G_{kl}+\varepsilon_{k},$$

where $X_{k1}∼N(62.4,11.5^{2})$ mimicking age and $X_{k2}∼\mathrm{Bernoulli}(0.52)$ mimicking sex (Lin *et al.* 2013). For each gene, we randomly selected 10 genetic variants assumed to influence the trait value. Additionally, we randomly chose 10 variants expected to affect the trait value through interaction with the environmental variable E. These 2 groups of variants could overlap. The environmental variable *E* is a Bernoulli random variable taking values of 0 or 1 with a probability of 0.5. The weight of a rare variant in $w_{1j}$ or $w_{2l}$ is set to Beta(MAF;1,25), the beta density function with parameters 1 and 25 evaluated at the variant's MAF, and the weight of a common variant in $w_{1j}$ or $w_{2l}$ is set to cBeta(MAF;0.5,0.5) with $c=\frac{\mathrm{Beta}(0.05;\;1,\;25)}{\mathrm{Beta}(0.05;\;0.5,\;0.5)}$ (Madsen and Browning 2009; Ionita-Laza *et al.* 2013). The error term $\varepsilon_{k}∼N(0,\;1.5^{2})$ indicates independent noise.

For a binary trait, we use the following logistic regression model


$$\begin{array}{rl} \mathrm{logit}(P(y_{k}=1))\;= & -6.2+0.05X_{k1}+0.057X_{k2}+0.64E_{k}+\overset{10}{\underset{\;j=1}{\sum }}\;w_{1j}\beta_{j}G_{kj}\\ & +\overset{10}{\underset{l=1}{\sum }}\;w_{2l}\gamma_{l}E_{k}G_{kl}, \end{array}$$

where all parameters are the same as those used in the continuous phenotype model. In every simulation scenario, each dataset includes 5,000 subjects (2,500 cases and 2,500 controls for binary phenotype).

### Simulation scenarios

In the type I error assessment, with all $\gamma_{l}$ set to 0 (indicating no G×E effects), we generated $10^{6}$ datasets, each containing 2 common and 8 rare variants from gene *A4GALT*. We tested the interaction effect between gene *A4GALT* and the environmental variable under 2 scenarios: (1) $\beta_{j}=0.05$ for common and 0.09 for rare variants and (2) the same $\beta_{j}$ values as in scenario (1), but with half of the common and rare variants having negative effects.

For the empirical power comparison, we tested the interaction effect between gene *A4GALT* and the environmental variable and repeated this procedure for gene *TIMP3*. Simulation scenarios differed in 3 key factors: (1) the selection method for the 10 variants in the genetic main effect and G×E interaction terms; (2) the directions of the genetic main effects $\beta_{j}$ and G×E interaction effects $\gamma_{l}$; and (3) the magnitude of the $\gamma_{l}$.

For factor 1, we considered 2 cases: (i) 10 variants comprising 2 common and 8 rare or (ii) any mix of common and rare variants, with variants chosen randomly in both cases. For factor 2, we considered 4 scenarios based on the signs of the $\beta_{j}$ and $\gamma_{l}$: (i) all positive $\beta_{j}$ and $\gamma_{l}$, (ii) 50% positive $\beta_{j}$ and all positive $\gamma_{l}$, (iii) all positive $\beta_{j}$ and 50% positive $\gamma_{l}$, and (iv) 50% positive $\beta_{j}$ and 50% positive $\gamma_{l}$. For factor 3, the magnitude of G×E interaction effects $\gamma_{l}$ was set to 0.19 for common variants and 0.49 or 0.59 for rare variants in continuous traits and 0.3 for common variants and 0.68 or 0.78 for rare variants in binary traits. The magnitude of the genetic main effects $\beta_{j}$ was set to 0.05 for common and 0.09 for rare variants across all simulation scenarios. In total, we considered 16 cases for each gene (*A4GALT* or *TIMP3*) and each trait type (continuous or binary): (2 selection methods) × (4 combinations of $\beta_{j}$ and $\gamma_{l}$ signs) × (2 $\gamma_{l}$ values for rare variants). This resulted in 64 scenarios.

In the 2 trait models, terms $0.05X_{k1}$, $0.057X_{k2}$, and $0.64E_{k}$ were set as in Lin *et al.* 2013. Remaining parameters, such as $\beta_{j}$, $\gamma_{l}$, and $\varepsilon_{k}$ are chosen to ensure that (1) the proportion of phenotypic variance explained by genetic variants ranges from 1.45 to 3.36% for the continuous trait; (2) the odds ratio and disease prevalence are around 0.078 and 0.088, respectively, for the binary trait; and (3) the G×E interaction effects are approximately 4–9 times greater than the genetic main effects.

### The MESA data

To demonstrate the utility of our proposed methods, we performed a genome-wide analysis of gene–alcohol interaction on hypertension and seated SBP in MESA (Bild *et al.* 2002). Heavy alcohol consumption is a well-known risk factor for hypertension (Simino *et al.* 2013). Interactions between genes and alcohol consumption are biologically plausible, with recent studies suggesting gene–alcohol interactions in blood pressure regulation (Lorenti Garcia *et al.* 2009; Alegría-Torres *et al.* 2011; Joenje 2011; Simino *et al.* 2013). MESA is a large ongoing longitudinal study of subclinical cardiovascular diseases (CVDs) including more than 6,800 participants. It uses a patient survey questionnaire to collect information on environmental and lifestyle factors (e.g. socioeconomic and psychosocial status, medical and family history, alcohol intake, and smoking). The data contains subclinical CVD variables such as cardiac output, coronary calcium, hypertension, blood pressure, and body mass index (BMI). We evaluated the incidence of hypertension during the initial physical examination of 6,403 participants, which included 2,851 individuals with hypertension and 3,552 without. Additionally, we assessed seated SBP in 6,425 participants at the same examination, with a median SBP of 123.5 mmHg. We adjusted the SBP measurements for participants on antihypertensive medication by following the standard procedure of adding 10 mmHg to their SBP (Cui *et al.* 2003; He *et al.* 2017). The study population represented a diverse group, comprising 39.3% white, 26.1% African American, 22.5% Hispanic, and 12.1% Asian participants. Alcohol usage (consumption of alcoholic beverages currently or formerly) was treated as an environmental variable, with 6,379 responses including 5,058 YESs and 1,321 NOs.

Samples were genotyped using the Affymetrix Human SNP Array 6.0. After data cleaning, imputation was performed using IMPUTE2 (Howie *et al.* 2009) with the 1000 Genome Phase 3 data as the reference panel. Subjects with less than 5% successfully imputed variants or empirical inbreeding coefficients greater than 0.05 were excluded, leaving 6,424 subjects for further analysis. The following quality-control criteria were applied: (1) call rate > 95%, (2) MAF > 0.5%, and (3) Hardy–Weinberg $\chi^{2}$ statistic *P*-value $>10^{-6}$, resulting in a final set of 8,540,864 variants. In the gene-based G×E analysis, we focused on protein-coding regions as defined by the reference genome GRCh37 (Frankish *et al.* 2019). This analysis covered 19,005 genes across the 22 chromosomes, with each gene region containing between 2 and 12,754 variants and a median of 384 variants per gene. After integrating data on hypertension, SBP, alcohol consumption, and genotype, the final sample sizes retained for downstream analyses were 6,375 individuals for hypertension and 6,373 for SBP.

### Pathway analysis

Functional pathway analysis of the G×E testing results for hypertension and SBP was performed using MetaCore software (Ekins *et al.* 2007). Genes with a *P*-value < 0.001 in at least 1 of the 5 tests were annotated and evaluated to identify enriched pathways associated with hypertension. This *P*-value threshold was chosen to yield a moderate-sized gene list, e.g. 40 genes in the hypertension study and 28 genes in the SBP analysis. The top genes were mapped to a reference set in MetaCore, and Fisher's exact test was applied to determine whether the gene list was significantly enriched in functional pathways than what would be expected by chance. Pathways with a false discovery rate (FDR) of <0.05 were considered significant.

## Results

### Type I error assessment and power comparison

The empirical type I error rate was calculated at nominal levels of α= 0.01, 0.001, and 0.0001, using 10^6^ replicates for both continuous and binary phenotypes across the 2 simulation scenarios (see Table 1). In most simulations, the type I errors for MAGEIT_RAN and MAGEIT_FIX were lower than the nominal levels, particularly for binary phenotype. This suggests that the MQS-based testing procedure tends to yield conservative *P*-values, likely due to the approximation methods used for handling binary phenotypes (Schweiger *et al.* 2017; Crawford and Zhou 2018).

**Table 1.** Empirical type I error of MAGEIT_RAN and MAGEIT_FIX, based on 10^6^ replicates.

Test	Nominal level	Continuous		Binary	
		Scenario 1	Scenario 2	Scenario 1	Scenario 2
MAGEIT_RAN	0.01	**1.07 × 10^−2^**	**9.21 × 10^−3^**	**9.50 × 10^−3^**	**9.15 × 10^−3^**
	0.001	**8.74 × 10^−4^**	**7.42 × 10^−4^**	**7.62 × 10^−4^**	**7.29 × 10^−4^**
	0.0001	**4.40 × 10^−5^**	**5.70 × 10^−5^**	**6.00 × 10^−5^**	**5.80 × 10^−5^**
MAGEIT_FIX	0.01	1.00 × 10^−2^	1.00 × 10^−2^	**9.49 × 10^−3^**	**9.47 × 10^−3^**
	0.001	**9.16 × 10^−4^**	**9.18 × 10^−4^**	**8.82 × 10^−4^**	**9.12 × 10^−4^**
	0.0001	**7.00 × 10^−5^**	**7.20 × 10^−5^**	9.70 × 10^−5^	9.60 × 10^−5^

Rates outside of the 95% confidence interval are in bold. The 95% confidence interval of a nominal level *α* was calculated as $\alpha \pm 1.96\sqrt{\alpha (1-\alpha )/10^{6}}$. Specifically, the 95% confidence intervals are $\left(9.80\times 10^{-3},\;1.02\times 10^{-2}\right)$ for α=0.01, $\left(9.38\times 10^{-4},\;1.06\times 10^{-3}\right)$ for α=0.001, and $\left(8.04\times 10^{-5},\;1.20\times 10^{-4}\right)$ for α=0.0001.

**Fig. 1.**
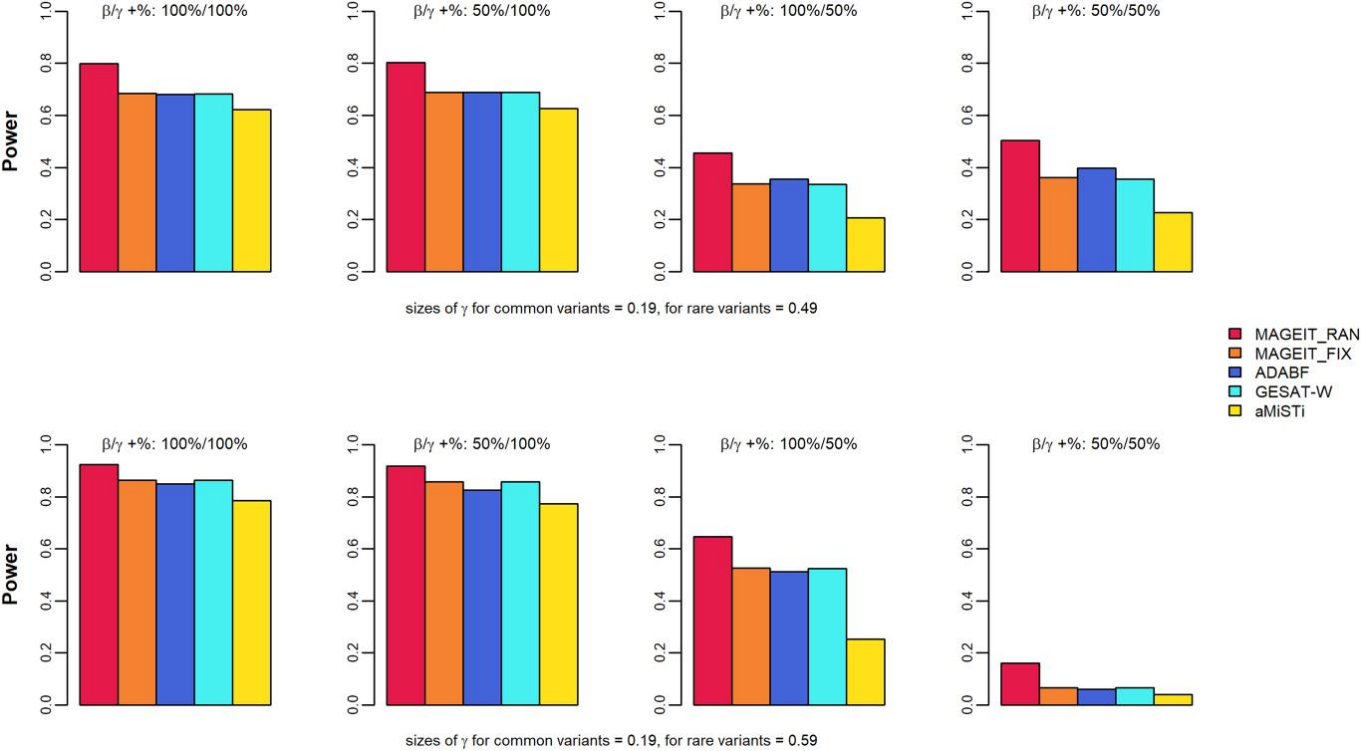
Empirical power of MAGIT_RAN, MAGIT_FIX, GESAT-W, aMiSTi, and ADABF for a continuous phenotype associated with the gene *A4GALT*. The gene set includes 2 common variants (MAF > 0.05) and 8 rare variants (0.005 < MAF < 0.05).

**Fig. 2.**
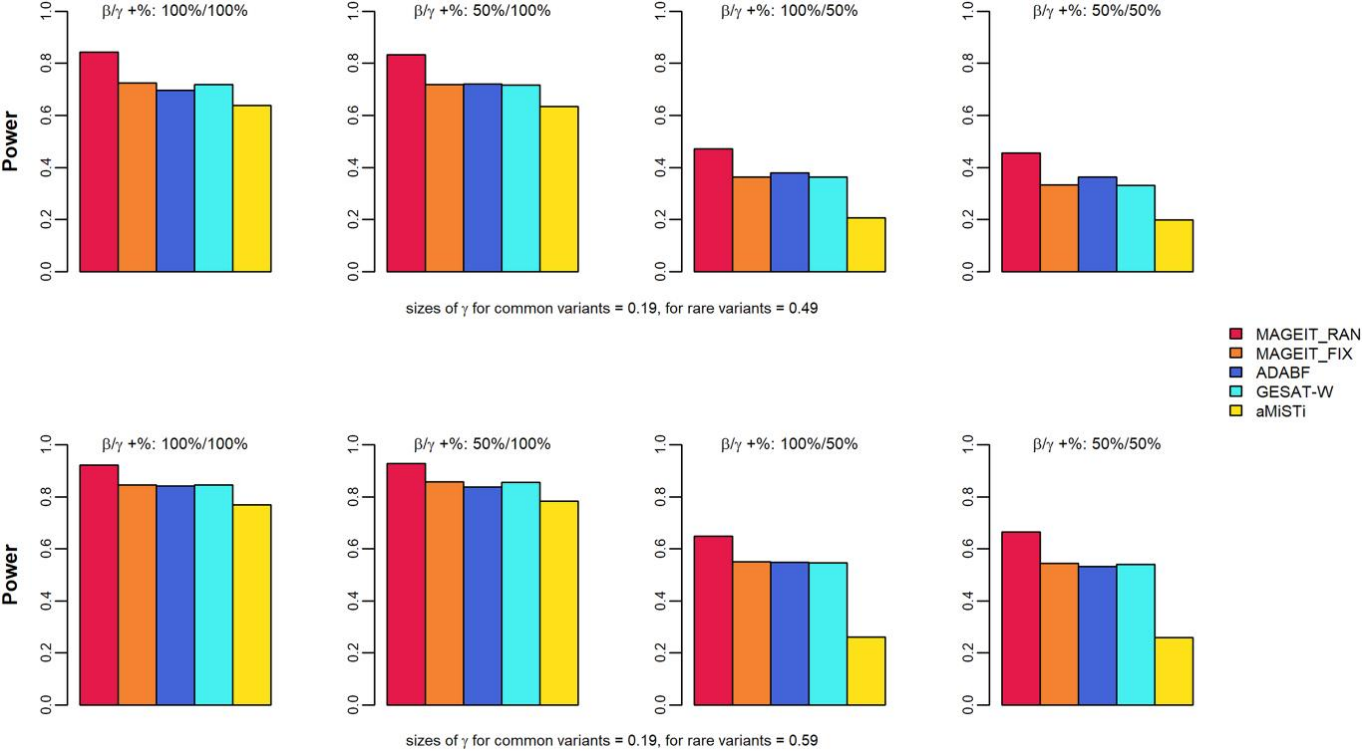
Empirical power of MAGIT_RAN, MAGIT_FIX, GESAT-W, aMiSTi, and ADABF for a continuous phenotype associated with the gene *A4GALT*. The gene set includes a random combination of common variants (MAF > 0.05) and rare variants (0.005 < MAF < 0.05).

**Fig. 3.**
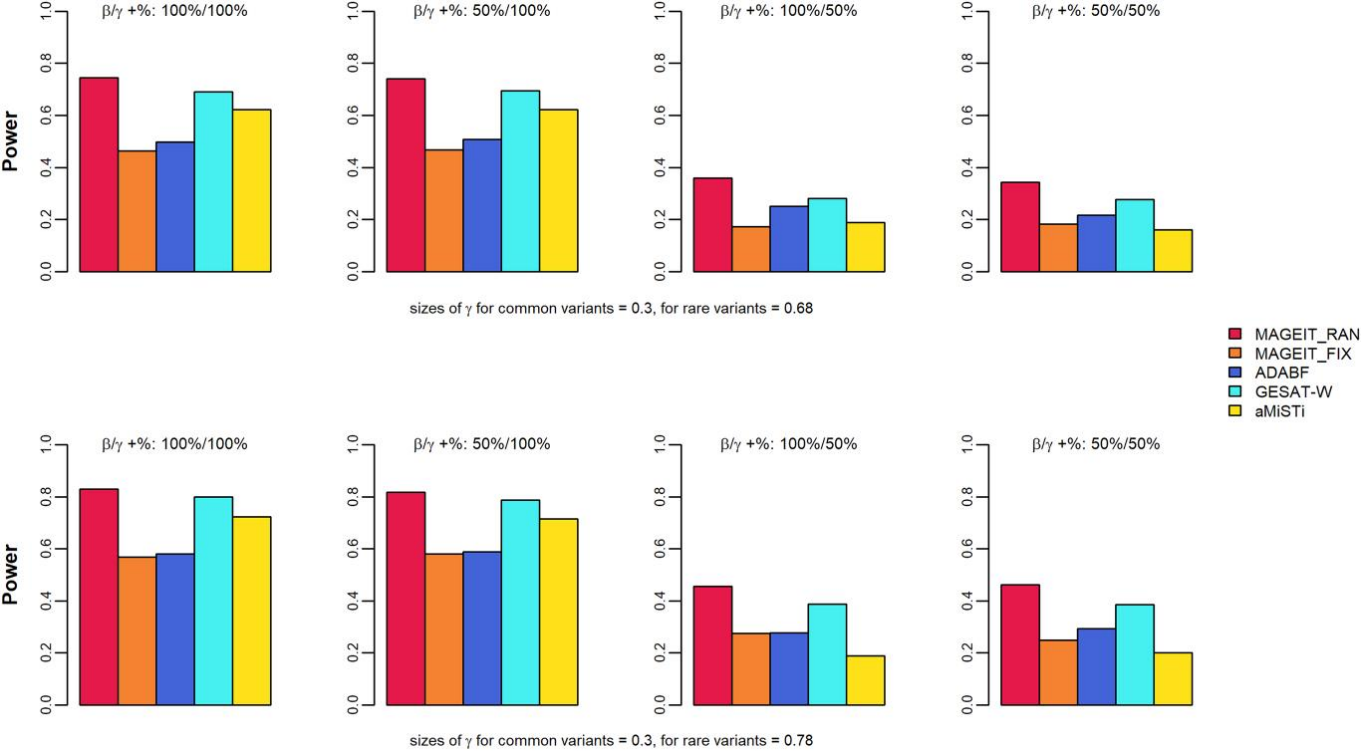
Empirical power of MAGIT_RAN, MAGIT_FIX, GESAT-W, aMiSTi, and ADABF for a binary phenotype associated with the gene *A4GALT*. The gene set includes 2 common variants (MAF > 0.05) and 8 rare variants (0.005 < MAF < 0.05).

**Fig. 4.**
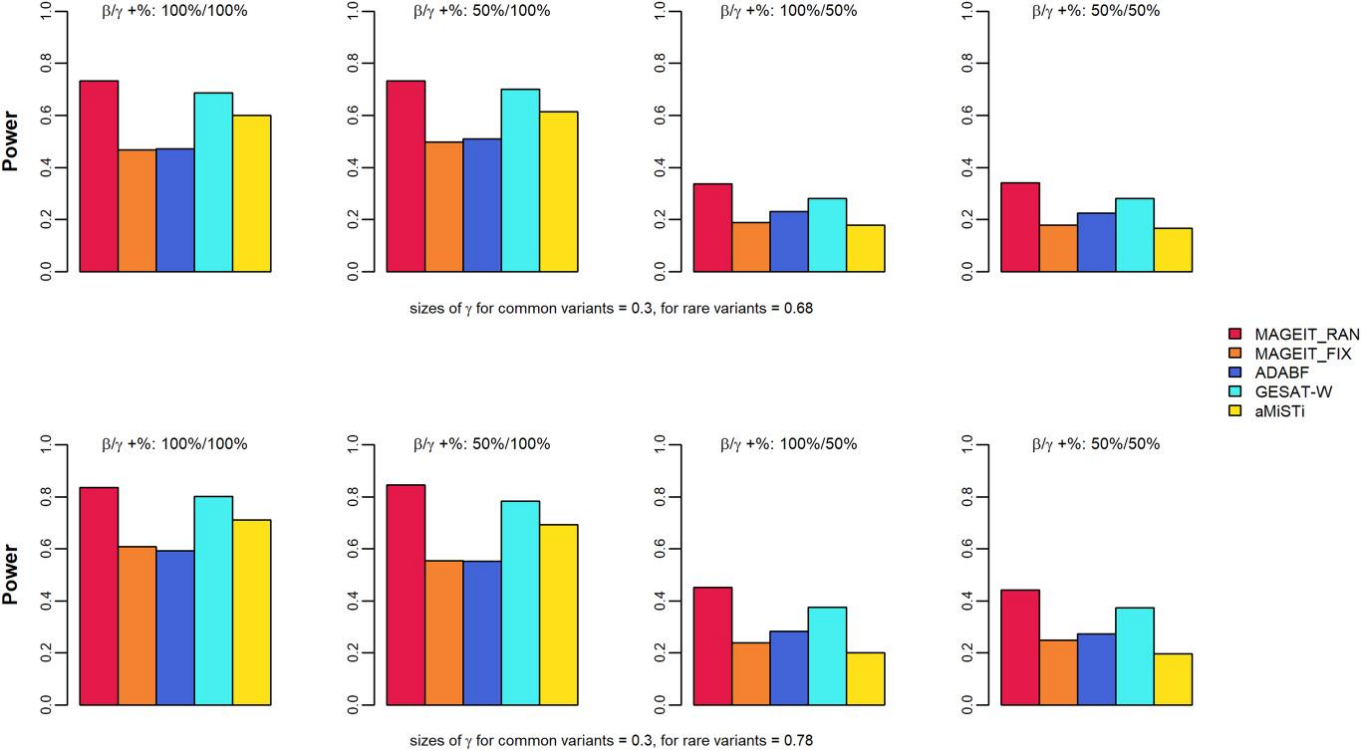
Empirical power of MAGIT_RAN, MAGIT_FIX, GESAT-W, aMiSTi, and ADABF for a binary phenotype associated with the gene *A4GALT*. The gene set includes a random combination of common variants (MAF > 0.05) and rare variants (0.005 < MAF < 0.05).

In genome-wide association studies, the significance threshold is typically much lower than 0.05 to account for multiple hypothesis testing. Therefore, the empirical power is evaluated at smaller nominal levels (Lin *et al.* 2016). In this study, empirical power was calculated at the significant level of $10^{-4}$, based on 1,000 simulation replicates. Figures 1 and 2 demonstrate the empirical power results of the 5 methods, MAGEIT_RAN, MAGEIT_FIX, GESAT-W, aMiSTi, and ADABF, under the 16 simulation cases for a continuous trait on the gene *A4GALT*. Specifically, the results corresponding to a random set of 2 common and 8 rare variants are given in Fig. 1. The top and bottom panels of Fig. 1 give the 2 scenarios where the G×E interaction effects $\gamma_{l}$ for rare variants are either 0.49 or 0.59, respectively. The 4 plots in each panel represent the 4 cases where the proportions of positive $\beta_{j}$ and positive $\gamma_{l}$ are 100%/100%, 50%/100%, 100%/50%, and 50%/50%. The results for a random set of common and rare variants are given in Fig. 2. Figures 3 and 4 give the empirical power results of the 16 cases for a binary trait on the gene *A4GALT*. Similar results for the gene *TIMP3* are presented in Supplementary Figs. 1–4 of the Supplemental Material. We also performed additional power simulations using MAF > 0.01 for common variants and 0.01 > MAF > 0.005 for rare variants. With these new MAF cutoff values, the gene *A4GALT* contains 200 variants, 110 (55%) common and 90 (45%) rare, while the gene *TIMP3* contains 296 variants, 199 (67%) common and 97 (33%) rare. The simulation results for these 2 genes, based on the new MAF cutoff values and the 2 trait types, are presented in Supplementary Figs. 5–12 of the Supplemental Material. Across all simulation scenarios, MAGEIT_RAN demonstrated the highest power compared to the other methods. The high power of MAGEIT_RAN may attribute to its unbiased and statistically efficient estimates of the variance component. Additionally, the genetic effects are treated as random in MAGEIT_RAN, which aligns with a more realistic assumption when the genetic region consists of both common and rare variants. MAGEIT_RAN is much more powerful than other tests when G×E effects include both positive and negative directions (as shown in the last 2 columns of all figures). For continuous traits, MAGEIT_FIX demonstrated comparable power to GESAT-W and ADABF and outperformed aMiSTi across all simulation scenarios. For binary phenotypes, however, GESAT-W demonstrated greater power compared to both MAGEIT_FIX and ADABF. However, it is important to note that the type I error rates of GESAT-W were inflated for binary traits across the 2 simulation scenarios (see Supplementary Table 1 in the Supplemental Material). When G×E effects had mixed positive and negative directions, aMiSTi showed the lowest power for both continuous and binary phenotypes. As a combination of burden and variance component tests, aMiSTi loses power when the genomic region being tested contains both protective and detrimental variants (Basu and Pan 2011).

### Analysis of G×E in MESA data

We conducted genome-wide tests to assess gene–alcohol interaction effects on hypertension and seated SBP using all 5 methods: MAGEIT_RAN, MAGEIT_FIX, GESAT-W, aMiSTi, and ADABF. The analysis included adjustments for age at the first exam, sex, and the top 10 principal components (PCs) of the genetic relationship matrix. These PCs were calculated using LD-pruned variants with an MAF > 0.05 to account for population structure.

In the hypertension analysis, MAGEIT_RAN and aMiSTi demonstrated no evidence of inflation, with the genomic control inflation factors of 0.974 and 0.966, respectively. The G×E test assuming fixed genetic main effects (MAGEIT_FIX) and the Bayes factor-based test (ADABF) were more conservative, showing genomic control inflation factors of 0.814 and 0.826, respectively. In contrast, GESAT-W had a genomic control inflation factor of 1.352, which can be partially attributed to its inflated type I error for binary traits (Supplementary Table 1). To address this, we adjusted the GESAT-W test statistic by dividing it by its genomic control inflation factor and then calculated the *P*-value of the adjusted statistic using the chi-squared distribution with 1 degree of freedom. In the SBP application, MAGEIT_RAN and aMiSTi again showed no inflation, with the genomic control inflation factors of 0.782 and 1.097, respectively. The genomic control inflation factors for MAGEIT_FIX, ADABF, and GESAT-W were approximately 1.474, and we adjusted their test statistics using the same procedure described above.

In the analysis of SBP, the gene *EIF2AK2* achieved genome-wide significance (*P*-value = $2.33\times 10^{-6}$) in gene-based analyses following Bonferroni correction (Epstein *et al.* 2015). This gene, displayed in Table 2 and identified by MAGEIT_RAN, is linked to pulse pressure via the SNP rs186895872, located 37 kb away (Sakaue *et al.* 2021). *EIF2AK2* is homologous to the PAH pathogenic gene *EIF2AK4*, which is a diagnostic marker for pulmonary venous occlusive disease (PVOD) (Eyries *et al.* 2014; Siddiqui and Charoenpong 2023). *EIF2AK2* encodes the protein kinase R (PKR) on chromosome 2, which animal studies have shown may affect hypertension through modulation of angiotensinogen and TGF-β, reducing fibrosis and apoptosis (Kalra *et al.* 2020). Also, research indicates that alcohol use disorders (AUD) are linked to a marked increase in PKR and p-PKR protein levels in the prefrontal cortex (PFC) (Johnson *et al.* 2015).

**Table 2.** Genes with genome-wide significance in the SBP analysis.

Chr	Gene	# SNP	Region	MAGEIT_RAN	MAGEIT_FIX	GESAT-W	aMiSTi	ADABF
2	*EIF2AK2*	370	2p22	**2.33 × 10^−6^**	1.31 × 10^−2^	1.42 × 10^−2^	2.87 × 10^−2^	4.64× 10^−2^

The smallest *P*-values among the 5 tests at the given genes are in bold.

In the study of hypertension, no genes reached genome-wide significance. Table 3 lists the 2 genes with the smallest and second-smallest *P*-values from at least one of the tests being compared. The gene *CCNDBP1* had the smallest *P*-value, identified by MAGEIT_RAN (*P*-value = $2.80\times 10^{-5}$), and the gene *EPB42* had the second lowest *P*-value, also identified by MAGEIT_RAN (*P*-value = $5.98\times 10^{-5}$). Both *CCNDBP1* and *EPB42* are located at 15q15.2. The cytogenetic region 15q15 has previously been reported to be associated with blood pressure (Kraja *et al.* 2005). Moreover, *EPB42* was shown to be significantly downregulated in heavy drinkers after being exposed to psychological stress (Beech *et al.* 2014; Chen *et al.* 2021; Ma *et al.* 2022).

**Table 3.** Genes with the top 2 lowest *P*-values in at least one of the tests in the hypertension analysis.

Chr	Gene	# SNP	Region	MAGEIT_RAN	MAGEIT_FIX	GESAT-W	aMiSTi	ADABF
15	*CCNDBP1*	237	15q15.2	**3.00 × 10^−5^**	1.03 × 10^−3^	4.83 × 10^−3^	2.97 × 10^−2^	4.70 × 10^−2^
	*EPB42*	269	15q15.2	**5.48 × 10^−5^**	1.34 × 10^−3^	5.58 × 10^−3^	2.93 × 10^−2^	6.30 × 10^−2^

The smallest *P*-values among the 5 tests at the given genes are in bold.

### Pathway analysis results

In the SBP application, we identified a significant apoptosis and survival pathway involving PKR's role in stress-induced apoptosis (*P*-value = $1.99\times 10^{-2}$, FDR = $3.16\times 10^{-2}$), at the false discovery rate (FDR) < 0.05. PKR activation in cardiac tissue is associated with inflammation and apoptosis, contributing to conditions such as hypertension, atherosclerosis, congestive heart failure (CHF), and stroke (Garcia *et al.* 2006). Additionally, studies have linked alcohol consumption to elevated PKR levels (Ke *et al.* 2011; Duncan *et al.* 2016).

In the analysis of hypertension, there are 2 significant signal transduction pathways that were reported to be related to hypertension. The first pathway is related to ERK1/2 signaling (*P*-value = $1.05\times 10^{-3}$, FDR = $2.67\times 10^{-2}$). ERK1/2 is instrumental in transmitting signals from the surface receptor to the nucleus. Once activated, it induces cell proliferation, differentiation, and other processes (Wortzel and Seger 2011). It has been reported that the ERK1/2-RSK-nNOS might be crucial in the regulation of central blood pressure influenced by Ang II (Cheng *et al.* 2010). The second pathway is a signal transduction pathway related to adenosine A1 receptor signaling (*P*-value = $3.89\times 10^{-3}$, FDR = $3.01\times 10^{-2}$). Adenosine modulates cardiovascular function and produces bradycardia and hypotension when mediated systematically (Barraco *et al.* 1987; Evoniuk *et al.* 1987). Activation of adenosine A1 receptor causes contraction of vascular smooth muscle and the adenosine A1 receptor agonists produce decreases in blood pressure and heart rate (Schindler *et al.* 2005). It has been observed that raised adenosine levels mediate the ataxic and sedative/hypnotic effects of ethanol through activation of A1 receptors in the cerebellum, striatum, and cerebral cortex (Ruby *et al.* 2010). A1 agonists have been shown to decrease anxiety-like behavior, tremor, and seizures during acute ethanol withdrawal in mice (Kaplan *et al.* 1999).

## Discussion

Human common diseases are shaped by a combination of genetic variations and interactions between genes and environmental factors. Numerous disease-associated genes have been successfully identified, making the understanding of gene–environment interactions essential for predicting disease risk (Hunter 2005). In this study, we developed 2 methods, MAGEIT_RAN and MAGEIT_FIX, to detect interactions between an environmental factor and a genetic marker set, where genetic main effects are modeled as random or fixed, respectively. Both methods are applicable to continuous and binary phenotypes. Our approach builds on the MQS estimation (Zhou 2017) with MAGEIT_RAN extending it by modeling genetic main effects as random. Given that variants within a genomic region can have both protective and detrimental effects with varying magnitudes, modeling genetic effects as random, as in MAGEIT_RAN, allows for the consideration of different directions and sizes of genetic effects.

We evaluated the performance of MAGEIT_RAN, MAGEIT_FIX, and 3 set-based G×E tests through extensive simulations. Our results showed that both methods maintained well-controlled type I error rates, with slightly conservative *P*-values. Across all simulation settings, MAGEIT_RAN demonstrated the highest overall statistical power among the 5 methods. While MAGIT_FIX exhibited comparable power to GESAT-W and ADABF for continuous traits, it showed reduced power for binary traits. Based on the findings from our simulation studies and real data applications, we recommend using MAGEIT_RAN for testing G×E interactions in gene sets that include both rare and common variants.

In the genome-wide analysis of gene–alcohol interactions on SBP using the MESA dataset, MAGEIT_RAN identified the gene *EIF2AK2* at a genome-wide significance level. This gene encodes the protein PKR, which has been associated with both hypertension and AUD. While no genes reached genome-wide significance in the hypertension analysis, MAGEIT_RAN yielded the 2 lowest *P*-values among the 5 methods tested and identified 2 genes, *CCNDBP1* and *EPB42*, located at the cytogenetic region 15q15.2, previously reported to be associated with blood pressure. The *EPB42* gene expression significantly downregulated in heavy drinkers following exposure to psychological stress. Furthermore, we identified 1 apoptosis and survival pathway involving PKR's role in stress-induced apoptosis. We also identified 2 signal transduction pathways related to hypertension, with 1 connected to both hypertension and alcohol use. These findings suggest that MAGEIT_RAN effectively identifies biologically relevant genes that interact with environmental factors to influence complex traits.

In MAGEIT_RAN, the regression coefficients for genetic main effects ($\beta_{j}$) and interaction effects ($\gamma_{j}$) are assumed to be independent. However, in reality, these effects may be correlated within a genomic region. This assumption could reduce statistical power, particularly when most variants in a gene interact with environmental factors in the same direction. Considering the complexities of linkage disequilibrium and haplotype effects, it may be more appropriate to account for potential correlations among these coefficients. In future work, we plan to extend the current model by incorporating correlations among variants within specific genomic regions to enhance the power of gene–environment interaction testing.

## Supplementary Material

jkae263_Supplementary_Data

## Data Availability

The MESA data can be downloaded from dbGaP (https://www.ncbi.nlm.nih.gov/gap/; Study Accession: phs000209.v13.p3) upon approval. Code to reproduce the results of the article is available at https://github.com/ZWang-Lab/MAGEIT2. Supplemental material available at G3 online.
