# Supplementary material for: Marginal interaction test for detecting interactions between genetic marker sets and environment in genome-wide studies
Source: G3 (Bethesda). 2024 Nov 14;15(1):jkae263. doi: 10.1093/g3journal/jkae263 (PMC11708225; doi:10.1093/g3journal/jkae263)
Supplement: jkae263_Supplementary_Data [file jkae263_supplementary_data.docx]

**Supplemental Material**

**
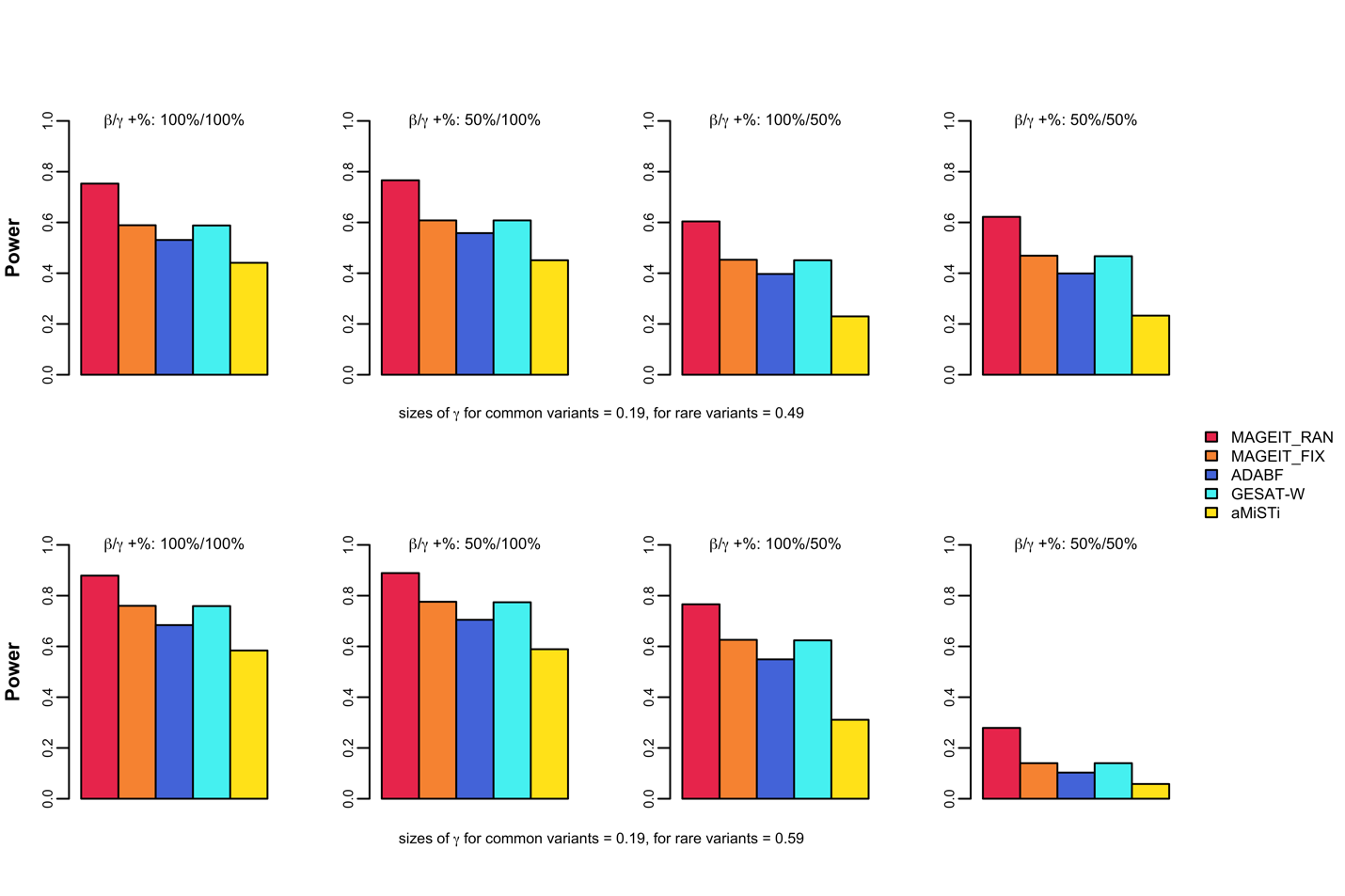
**

**Figure S1.** Empirical power of MAGIT_RAN, MAGIT_FIX, GESAT-W, aMiSTi, and ADABF for a continuous phenotype associated with the gene *TIMP3*. The gene set includes two common variants (MAF > 0.05) and eight rare variants (0.005 < MAF < 0.05).

**
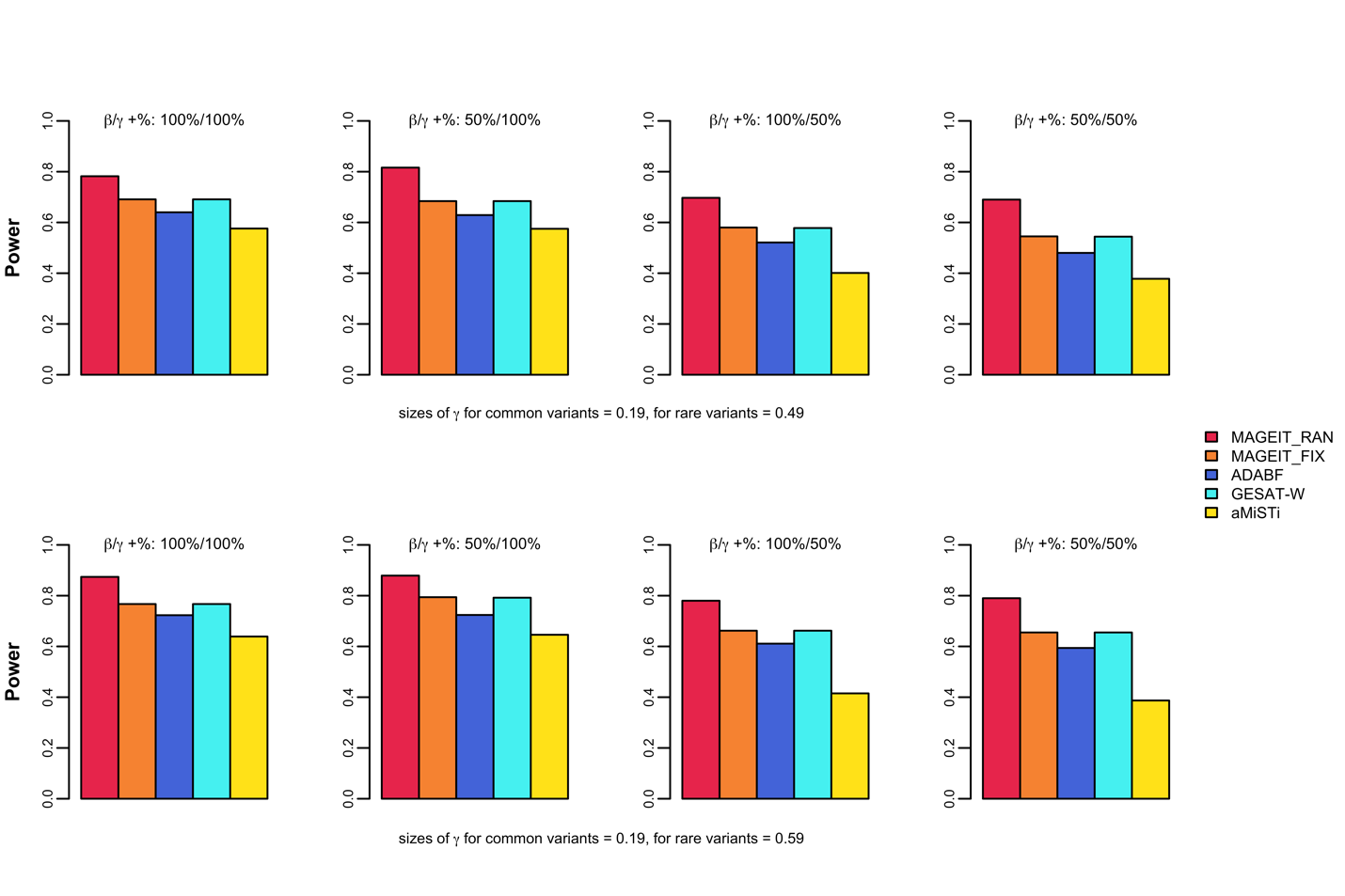
**

**Figure S2.** Empirical power of MAGIT_RAN, MAGIT_FIX, GESAT-W, aMiSTi, and ADABF for a continuous phenotype associated with the gene *TIMP3*. The gene set includes a random combination of common variants (MAF > 0.05) and rare variants (0.005 < MAF < 0.05).

**
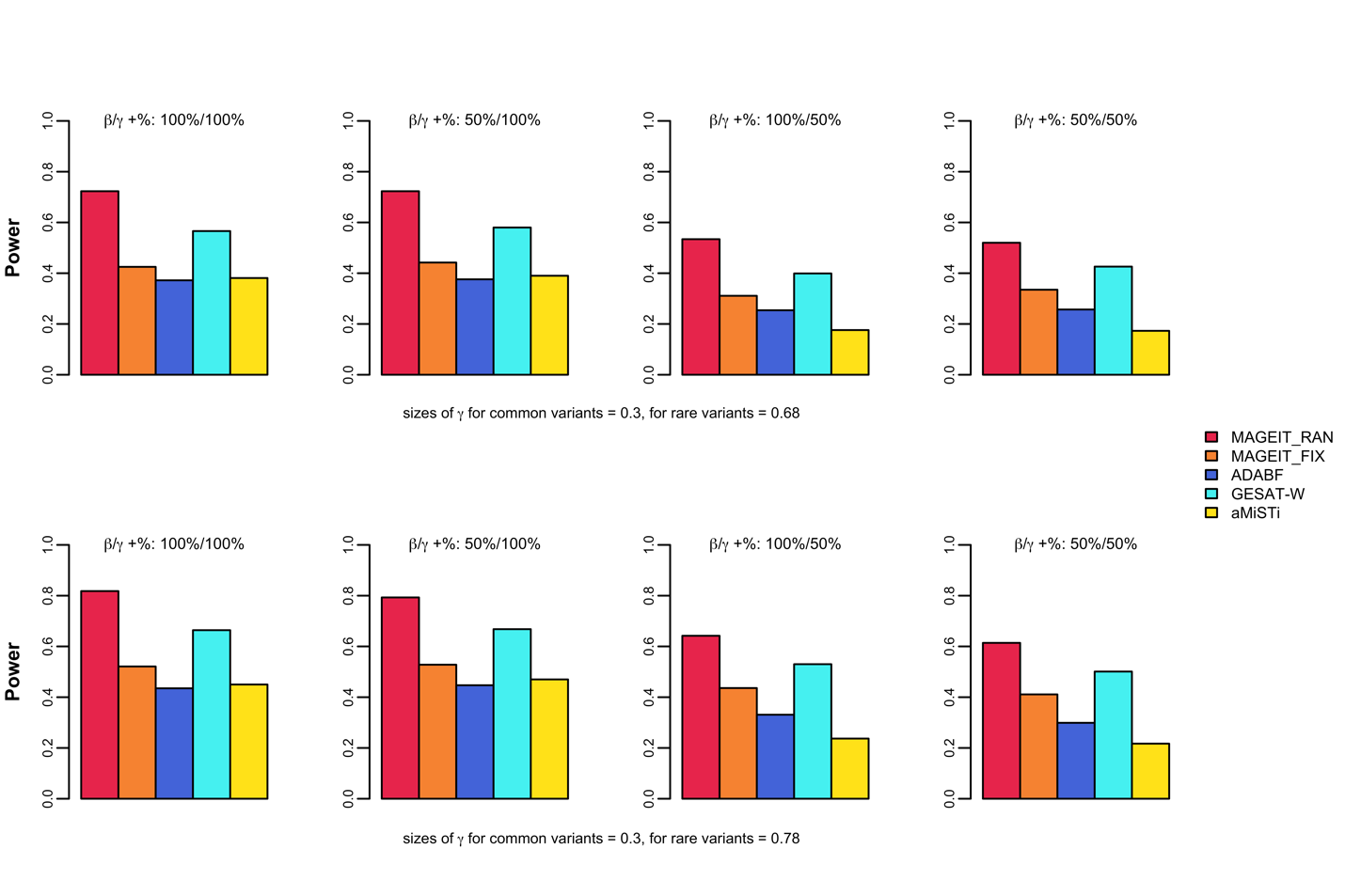
**

**Figure S3.** Empirical power of MAGIT_RAN, MAGIT_FIX, GESAT-W, aMiSTi, and ADABF for a binary phenotype associated with the gene *TIMP3*. The gene set includes two common variants (MAF > 0.05) and eight rare variants (0.005 < MAF < 0.05).

**
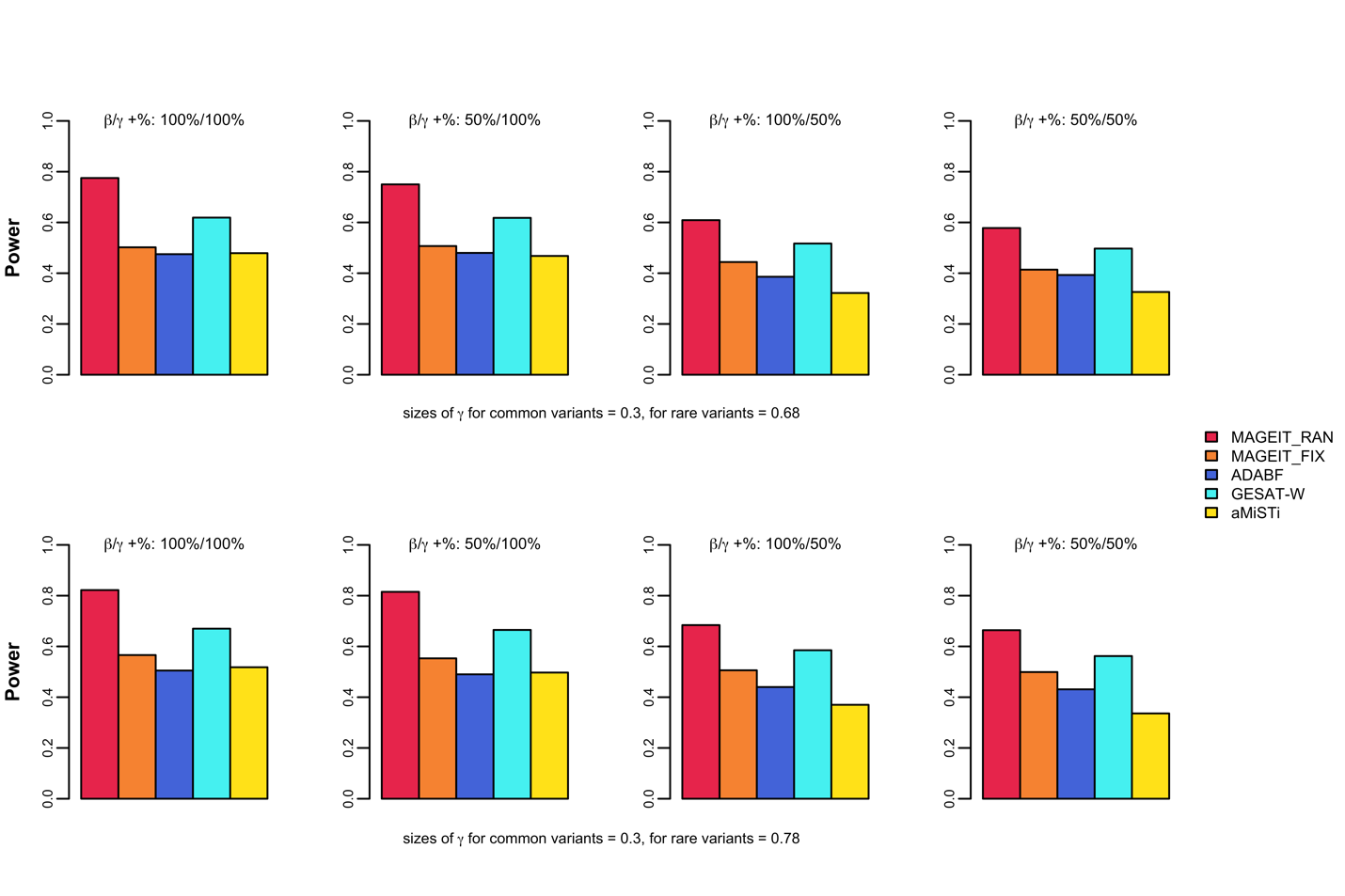
**

**Figure S4.** Empirical power of MAGIT_RAN, MAGIT_FIX, GESAT-W, aMiSTi, and ADABF for a binary phenotype associated with the gene *TIMP3*. The gene set includes a random combination of common variants (MAF > 0.05) and rare variants (0.005 < MAF < 0.05).

**
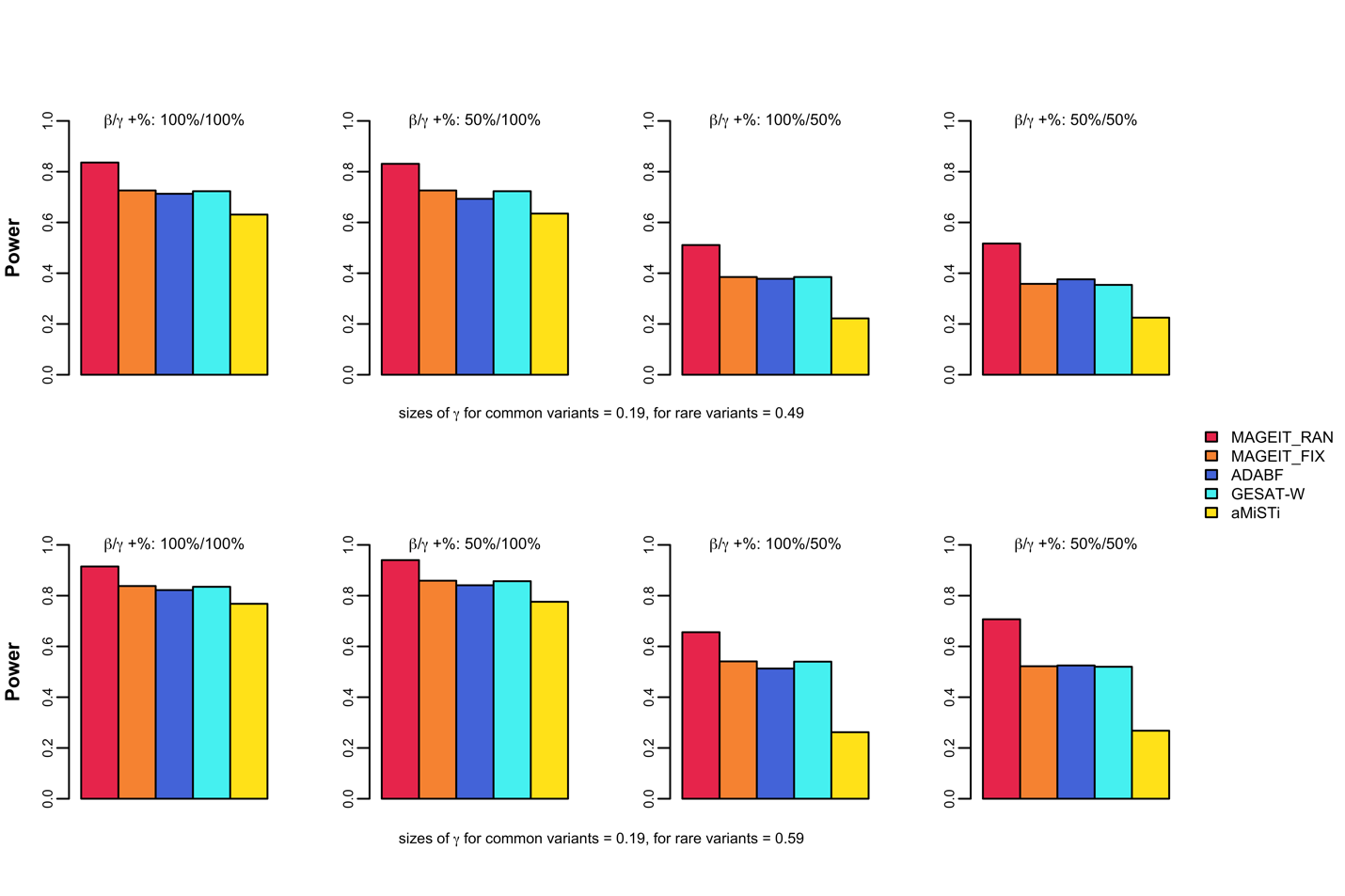
**

**Figure S5.** Empirical power of MAGIT_RAN, MAGIT_FIX, GESAT-W, aMiSTi, and ADABF for a continuous phenotype associated with the gene *A4GALT*. The gene set includes two common variants (MAF > 0.01) and eight rare variants (0.005 < MAF < 0.01).

**
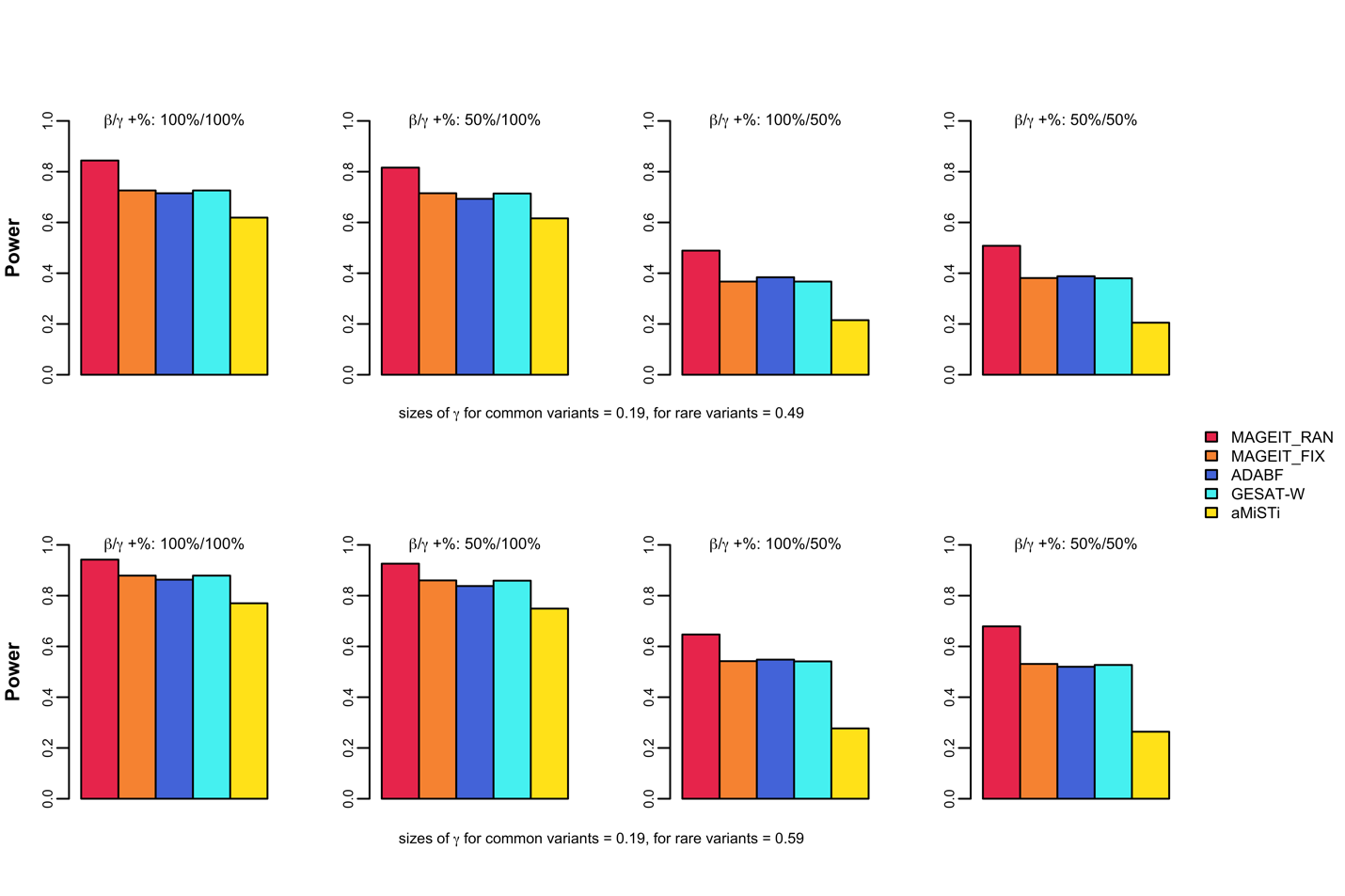
**

**Figure S6.** Empirical power of MAGIT_RAN, MAGIT_FIX, GESAT-W, aMiSTi, and ADABF for a continuous phenotype associated with the gene *A4GALT*. The gene set includes a random combination of common variants (MAF > 0.01) and rare variants (0.005 < MAF < 0.01).

**
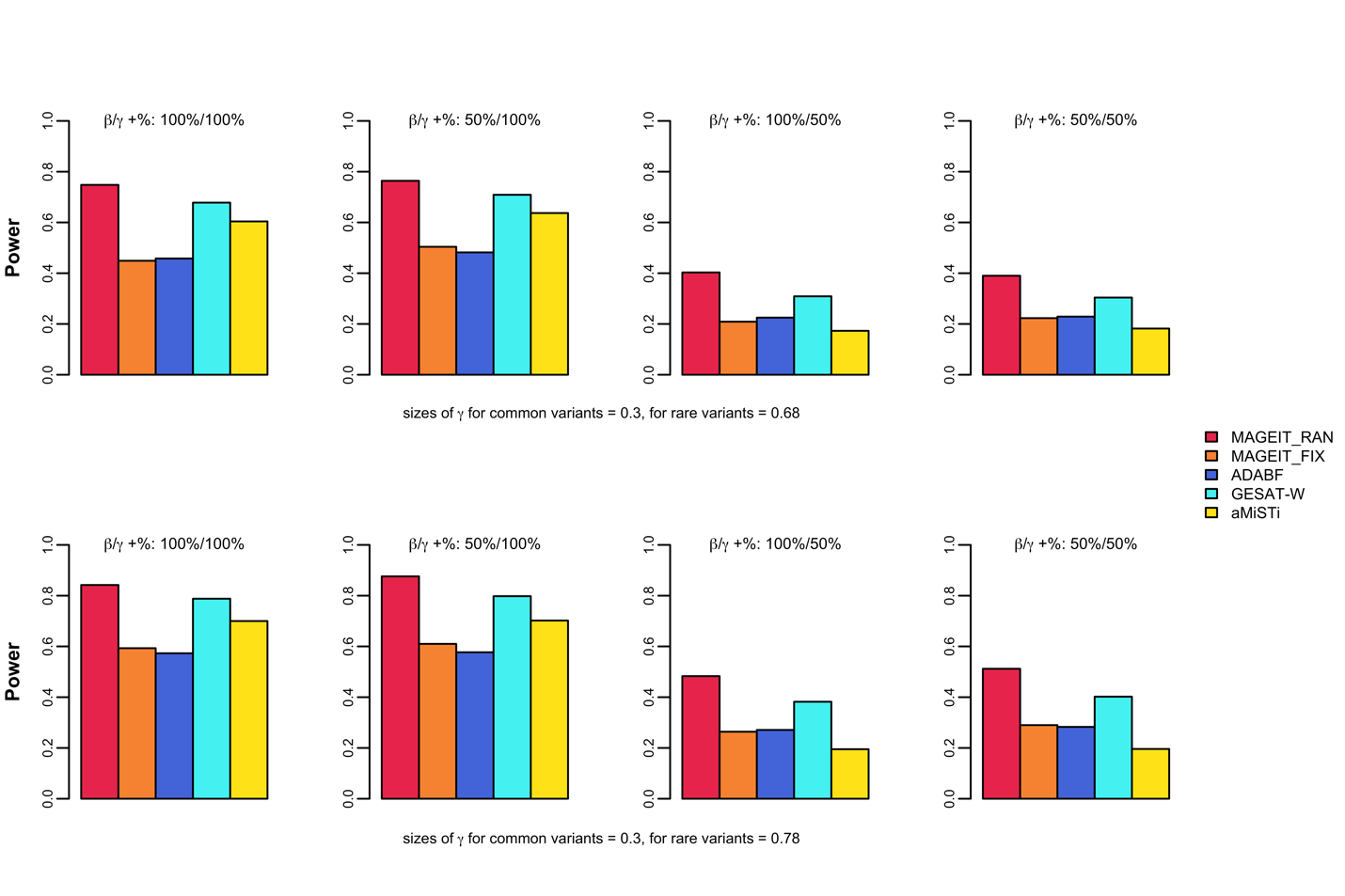
**

**Figure S7.** Empirical power of MAGIT_RAN, MAGIT_FIX, GESAT-W, aMiSTi, and ADABF for a binary phenotype associated with the gene *A4GALT*. The gene set includes two common variants (MAF > 0.01) and eight rare variants (0.005 < MAF < 0.01).

**
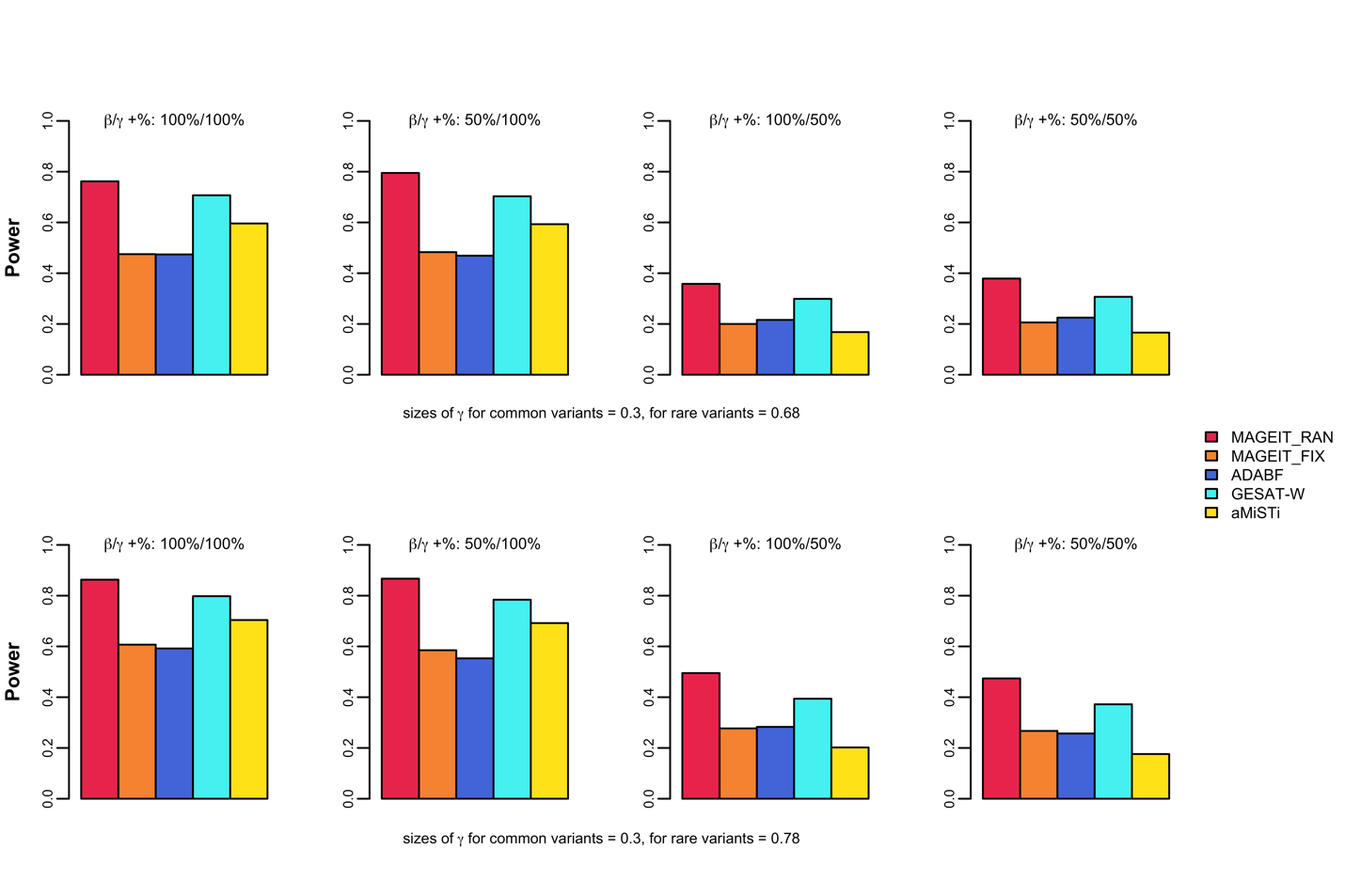
**

**Figure S8.** Empirical power of MAGIT_RAN, MAGIT_FIX, GESAT-W, aMiSTi, and ADABF for a binary phenotype associated with the gene *A4GALT*. The gene set includes a random combination of common variants (MAF > 0.01) and rare variants (0.005 < MAF < 0.01).

**
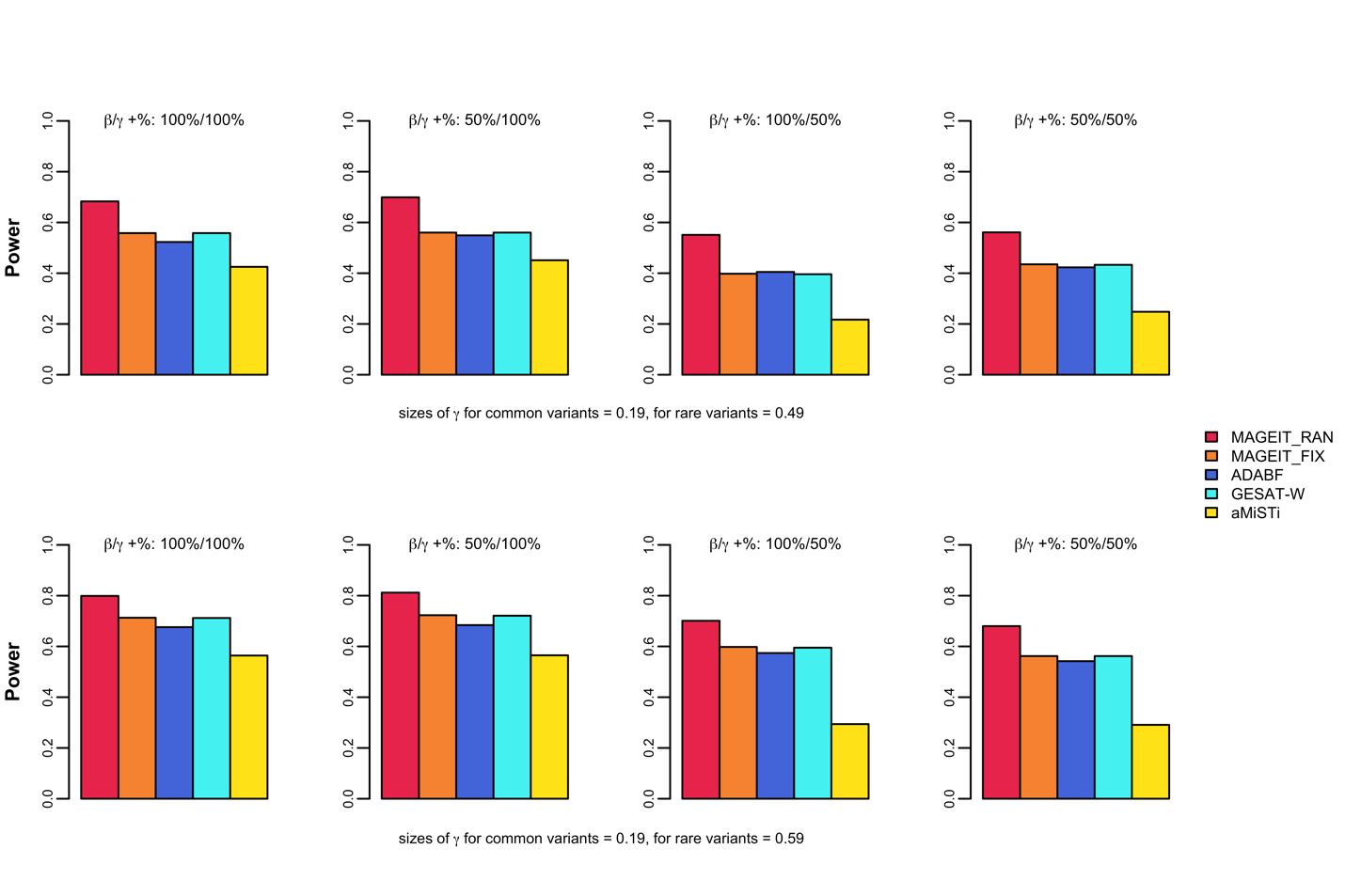
**

**Figure S9.** Empirical power of MAGIT_RAN, MAGIT_FIX, GESAT-W, aMiSTi, and ADABF for a continuous phenotype associated with the gene *TIMP3*. The gene set includes two common variants (MAF > 0.01) and eight rare variants (0.005 < MAF < 0.01).

**
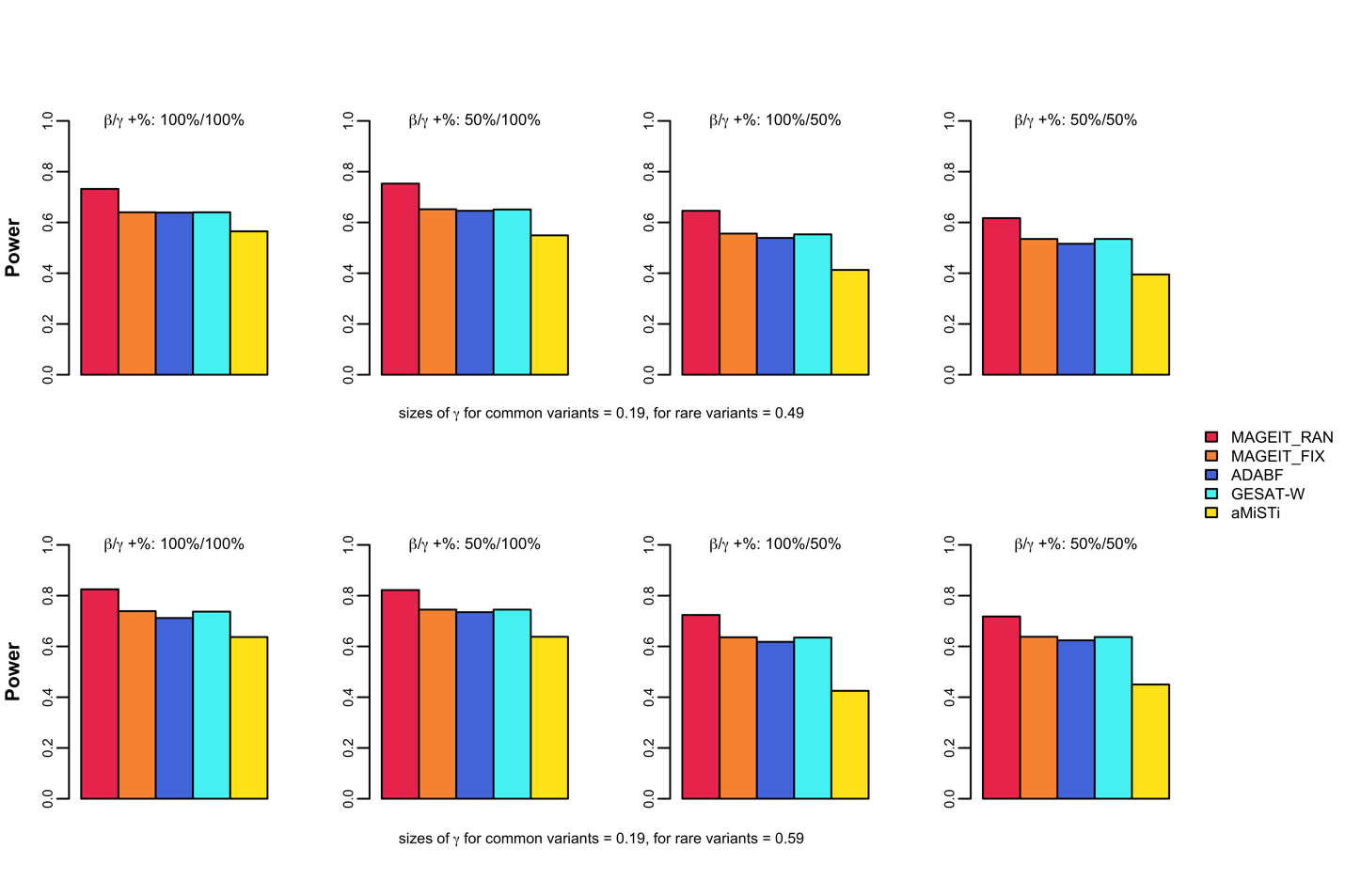
**

**Figure S10.** Empirical power of MAGIT_RAN, MAGIT_FIX, GESAT-W, aMiSTi, and ADABF for a continuous phenotype associated with the gene *TIMP3*. The gene set includes a random combination of common variants (MAF > 0.01) and rare variants (0.005 < MAF < 0.01).

**
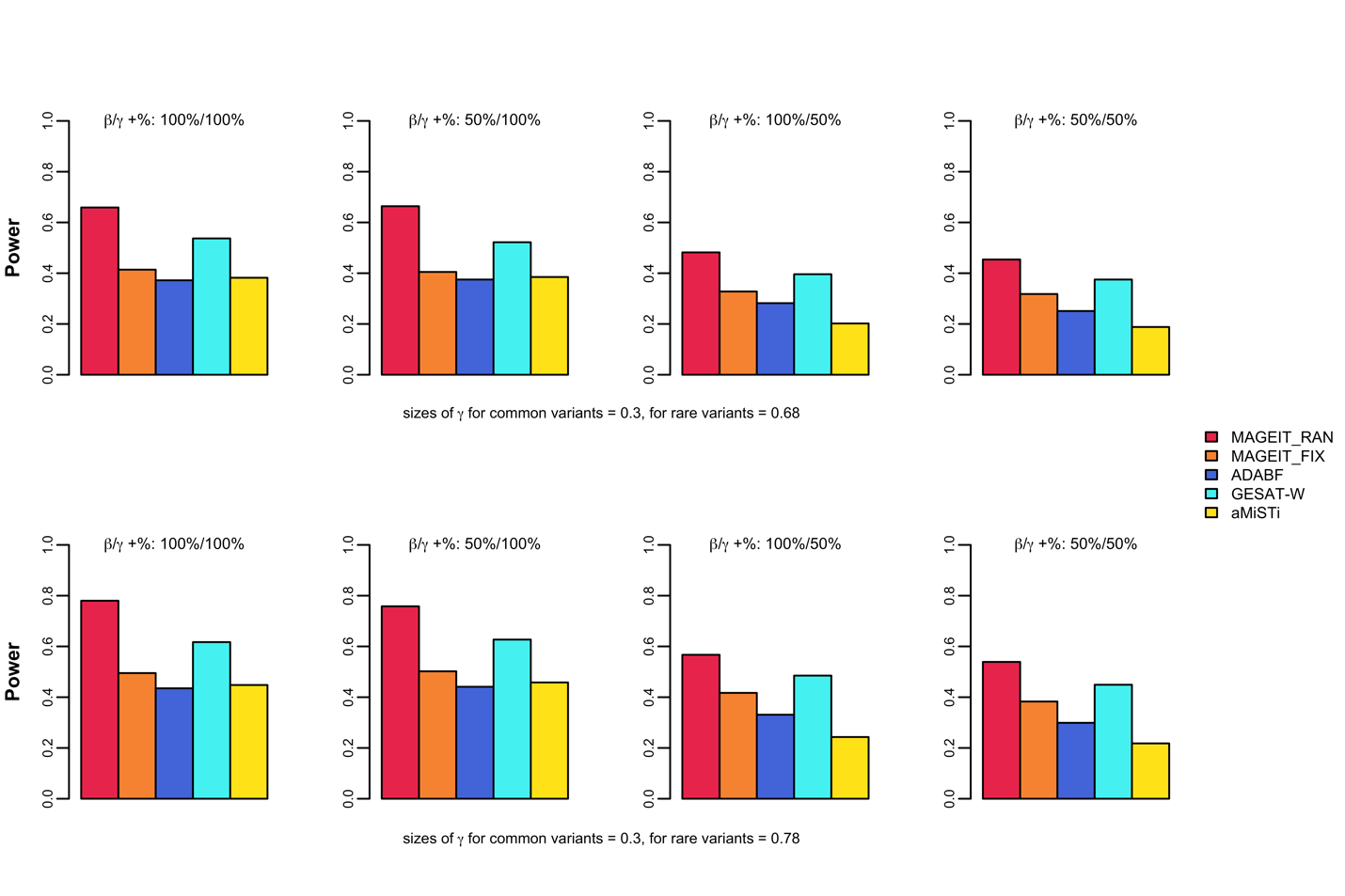
**

**Figure S11.** Empirical power of MAGIT_RAN, MAGIT_FIX, GESAT-W, aMiSTi, and ADABF for a binary phenotype associated with the gene *TIMP3*. The gene set includes two common variants (MAF > 0.01) and eight rare variants (0.005 < MAF < 0.01).

**
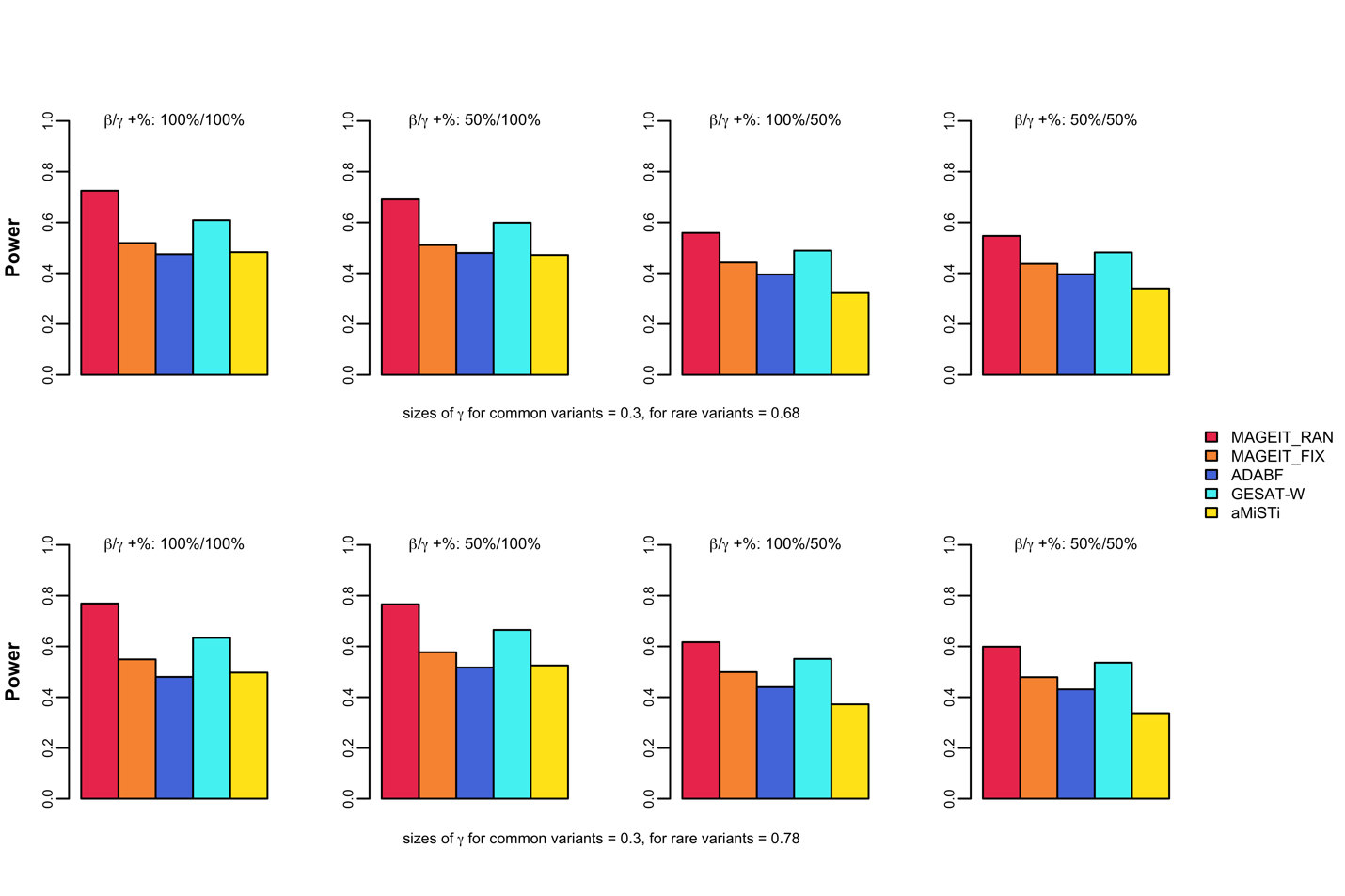
**

**Figure S12.** Empirical power of MAGIT_RAN, MAGIT_FIX, GESAT-W, aMiSTi, and ADABF for a binary phenotype associated with the gene *TIMP3*. The gene set includes a random combination of common variants (MAF > 0.01) and rare variants (0.005 < MAF < 0.01).

**Table S1.** Empirical type I error of GESAT-W, based on 10^6^ replicates

| **Test** | **Nominal**  **Level** | **Continuous** | | **Binary** | |
| --- | --- | --- | --- | --- | --- |
|  |  | **Scenario 1** | **Scenario 2** | **Scenario 1** | **Scenario 2** |
| GESAT | 0.01 | 9.82 × 10^-3^ | 9.82 × 10^-3^ | **2.63 × 10^-2^** | **2.66 × 10^-2^** |
|  | 0.001 | 9.41 × 10^-4^ | 1.01 × 10^-3^ | **3.38 × 10^-3^** | **3.48 × 10^-3^** |
|  | 0.0001 | 8.30 × 10^-5^ | 1.06 × 10^-4^ | **4.70 × 10^-4^** | **4.45 × 10^-4^** |

Rates outside of the 95% confidence interval are in bold. The 95% confidence interval of a nominal level $\alpha$ was calculated as $\alpha\pm1.96\sqrt{\alpha(1-\alpha)/{10}^{6}}$. Specifically, the 95% confidence intervals are $\left( 9.80\times{10}^{-3}, 1.02\times{10}^{-2} \right)$ for $\alpha=0.01$, $(9.38\times{10}^{-4}, 1.06\times{10}^{-3})$ for $\alpha=0.001$, and $(8.04\times{10}^{-5}, 1.20\times{10}^{-4})$ for $\alpha=0.0001$.
